# Blockade of dopamine D3 receptors improves hippocampal synaptic function and rescues age‐related cognitive phenotype

**DOI:** 10.1111/acel.14291

**Published:** 2024-09-05

**Authors:** Maria Rosaria Tropea, Marcello Melone, Domenica Donatella Li Puma, Valeria Vacanti, Giuseppe Aceto, Bruno Bandiera, Roberta Carmela Trovato, Sebastiano Alfio Torrisi, Gian Marco Leggio, Agostino Palmeri, Marcello D'Ascenzo, Fiorenzo Conti, Claudio Grassi, Daniela Puzzo

**Affiliations:** ^1^ Department of Biomedical and Biotechnological Sciences University of Catania Catania Italy; ^2^ Section of Neuroscience and Cell Biology, Department of Experimental and Clinical Medicine Università Politecnica Delle Marche Ancona Italy; ^3^ Center for Neurobiology of Aging, IRCCS INRCA Ancona Italy; ^4^ Department of Neuroscience Università Cattolica del Sacro Cuore Rome Italy; ^5^ Fondazione Policlinico Universitario A. Gemelli IRCCS Rome Italy; ^6^ Oasi Research Institute‐IRCCS Troina Italy

**Keywords:** aging, dopamine D3 receptors, hippocampus, memory, synaptic plasticity

## Abstract

Dopamine D3 receptors (D3Rs) modulate neuronal activity in several brain regions including the hippocampus. Although previous studies reported that blocking D3Rs exerts pro‐cognitive effects, their involvement in hippocampal synaptic function and memory in the healthy and aged brain has not been thoroughly investigated. We demonstrated that in adult wild type (WT) mice, D3R pharmacological blockade or genetic deletion as in D3 knock out (KO) mice, converted the weak form of long‐term potentiation (LTP1) into the stronger long‐lasting LTP (LTP2) via the cAMP/PKA pathway, and allowed the formation of long‐term memory. D3R effects were mainly mediated by post‐synaptic mechanisms as their blockade enhanced basal synaptic transmission (BST), AMPAR‐mediated currents, mEPSC amplitude, and the expression of the post‐synaptic proteins PSD‐95, phospho(p)GluA1 and p‐CREB. Consistently, electron microscopy revealed a prevalent expression of D3Rs in post‐synaptic dendrites. Interestingly, with age, D3Rs decreased in axon terminals while maintaining their levels in post‐synaptic dendrites. Indeed, in aged WT mice, blocking D3Rs reversed the impairment of LTP, BST, memory, post‐synaptic protein expression, and PSD length. Notably, aged D3‐KO mice did not exhibit synaptic and memory deficits. In conclusion, we demonstrated the fundamental role of D3Rs in hippocampal synaptic function and memory, and their potential as a therapeutic target to counteract the age‐related hippocampal cognitive decline.

## Graphical abstract

Dopamine D3 receptors (D3Rs) blockade acts through the cAMP/PKA pathway leading to long‐lasting LTP and memory. D3Rs are mainly located in post‐synaptic dendrites and their blockade enhances basal synaptic transmission, AMPAR‐mediated currents, mEPSC amplitude, and the expression of PSD‐95, pGluA1, pCREB. In aged mice, D3R blockade reversed synaptic transmission and plasticity, post‐synaptic protein expression, and PSD length. Aged D3‐KO mice did not exhibit synaptic and memory deficits.

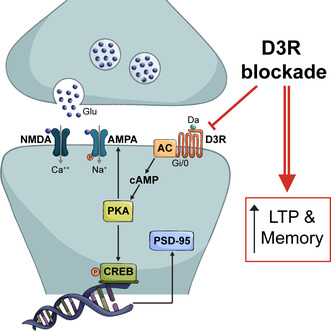

**Abbreviations**
AC: adenylyl cyclase
ACSF: artificial cerebrospinal fluid
AL: adjacent left quadrant
AMPAR: α‐amino‐3‐hydroxy‐5‐methyl‐4‐isoxazolepropionic acid receptor
ANOVA: analysis of Variance
APV: (2R)‐amino‐5‐phosphonovaleric acid
AR: adjacent right quadrant
ARRIVE: Animal Research: Reporting of In Vivo Experiments
AsP: proximal glial processes
Astro: astrocytes
Ax: axons
AxT: axon terminals
BCA: bicinchoninic acid
BST: basal synaptic transmission
CA1: Cornu Ammonis area 1
CA3: Cornu Ammonis area 3
cAMP: cyclic adenosine monophosphate
CREB: cAMP Response Element‐Binding Protein
Cyt: cytoplasm
D: discrimination index
D2R: dopamine D2 type receptor
D3‐KO: dopamine D3 type receptor knock out
D3R: dopamine D3 type receptor
Da: dopamine
DAB: 3,3'‐Diaminobenzidine
Den: dendrites
DMSO: dimethyl sulfoxide
ECL: enhanced chemiluminescence
EM: Electron Microscopy
EPSC: Excitatory Post‐Synaptic Current
fEPSP: field Excitatory Post‐Synaptic Potentials
GABA: gamma‐aminobutyric acid
GAPDH: glyceraldehyde 3‐phosphate dehydrogenase
Glu: glutamate
GluA1: AMPAR subunit glutamate receptor 1
ih: intrahippocampal
ip: intraperitoneal
IR: immunoreactivity
KO: knock out
LTM: long‐term Memory
LTP: long‐term potentiation
mEPSC: miniature EPSC
mRNA: messenger ribonucleic acid
MW: molecular weight
MWM: Morris Water Maze
Neu: neurons
NMDAR: N‐Methyl‐D‐Aspartate receptor
NOL: novel object location
NOR: Novel object recognition
OF: open field
OQ: opposite quadrant
PaP: pre‐synaptic astrocytic processes
p‐CREB: phospho‐CREB (Ser133)
p‐GluA1: phospho‐GluA1 (Ser 845)
PKA: protein kinase A
Post den: post‐synaptic dendrites
PPF: paired pulse facilitation
PSD: post‐synaptic density
PSD‐95: post‐synaptic density protein 95
PTP: post‐tetanic potentiation
pyr: stratum pyramidalis
Rp‐8‐Br‐cAMPS: Rp‐8‐Bromoadenosine‐3',5'‐cyclic monophosphorothioat
SDS: sodium dodecyl sulfate
SDS‐PAGE: SDS Polyacrylamide Gel Electrophoresis
SEM: Standard Error Mean
so: stratum oriens
sr: stratum radiatum
T1: training phase
T2: testing phase
TBS: Theta Burst Stimulation
TQ: target quadrant
Tris‐HCl: Tris(hydroxymethyl)aminomethane Hydrochloride
WB: western blot
WT: wild type

## 1 INTRODUCTION

Dopamine plays a crucial role in various cognitive functions, including memory, and alterations in dopaminergic transmission have been associated with age‐related cognitive decline (Berry et al., 2016; Hemby et al., 2003; Kaasinen et al., 2000; Shohamy & Wimmer, 2013). In this context, we focused on the metabotropic dopamine D3 receptors (D3Rs) belonging to the D2‐like family, which are negatively coupled to adenylyl cyclase (AC), resulting in a reduction of cAMP levels and the inhibition of PKA (Robinson & Caron, 1997). Despite being less abundant than the D2 subtype, dopamine exhibits a higher affinity for D3Rs rather than D2Rs (reviewed in Maramai et al., 2016), so even slight modifications in their function can result in significant changes in synaptic transmission, making them an intriguing target for pharmacological interventions. Therefore, they have been investigated in the pathophysiology and treatment of schizophrenia, depression, drug addiction, and locomotor disorders (reviewed in Kiss et al., 2021).

D3Rs are expressed in different brain areas, including the hippocampus (Li & Kuzhikandathil, 2012; Meador‐Woodruff et al., 1994), which is mainly involved in memory formation and consolidation. Both a direct and indirect interaction between D3Rs and glutamatergic synapses has been demonstrated, suggesting their role in the modulation of excitatory signals (Sokoloff et al., 2013). However, their contribution to hippocampal synaptic plasticity and memory in the healthy brain and during aging is not entirely understood. Previous studies demonstrated that a selective pharmacological blockade of D3Rs improved social and recognition memory (Millan et al., 2007; Sigala et al., 1997; Watson et al., 2012), and that D3R genetic deletion enhanced cognitive functions (Leggio et al., 2021; Micale et al., 2010; Xing et al., 2010). Similarly, in humans, activation of D3Rs impaired cognitive performance, whereas their blockade exerted cognitive enhancing effects (Gross et al., 2013).

Based on these findings, here we investigated the role of D3Rs in modulating hippocampal excitatory synaptic function and cognition in the healthy brain and during aging in mouse models. We have studied the effects of the pharmacological blockade or genetic deletion of D3Rs, and whether targeting of D3Rs could offer potential avenues for addressing age‐related cognitive decline. Furthermore, we have conducted an electron microscopic study of the CA1 area of the hippocampus to analyze the subcellular and synaptic distribution of D3Rs.

## 2 MATERIALS AND METHODS

### 2.1 Animals

We used WT mice (C57BL/6 J; RRID:IMSR_JAX:000664) purchased from The Jackson Laboratory. D3R−/− mice (D3‐KO) were generated by Accili et al. (1996) and subsequently backcrossed to C57BL/6J mice to obtain a congenic colony after 10th–12th generations. Colonies were established in the animal facilities at University of Catania, Università Politecnica delle Marche and Università Cattolica del Sacro Cuore. The housing conditions were controlled, maintaining stable hygrometric and thermic conditions (50%; 21°C ± 1°C) on a 12‐h light/dark cycle with ad libitum access to food and water.

Animal care, handling, and procedures were carried out in accordance with national and European Community Council Directives (2010/63/UE) and were approved by the Italian Ministry of Health (226/2021‐PR, 40A31.N.ZUK, 623/2022‐PR). The experiments complied with the ARRIVE guidelines and were conducted to minimize animal suffering.

We used sex‐balanced mice for electrophysiological recordings, behavioral experiments, and western blotting, and male mice for electron microscopy. Animals were used at 4–8 months (adult) and 18–22 months (old) according to our scientific work plan.

### 2.2 Drugs

Hippocampal slices were treated with NGB‐2904 (Sigma‐Aldrich, 1 μM), SB‐277011A (Sigma‐Aldrich, 10 μM), NKY‐80 (Abcam, 10 μM), and Rp‐8‐Br‐cAMPS (Biolog, 20 μM). NGB‐2904 and Rp‐8‐Br‐cAMPS were dissolved in DMSO, whereas other drugs in bidistilled water. Aliquots were diluted in ACSF to the desired final concentration right before electrophysiological experiments. For behavioral studies, we dissolved NGB‐2904 in a solution containing DMSO and Tween 20, and then diluted in saline solution (0.9% NaCl) to a dose of 3 mg/kg in a final volume of 200 μL for intraperitoneal (ip) administration. This dose was selected according to a previous work showing a pro‐cognitive effect of NGB‐2904 (Huang et al., 2015).

### 2.3 Electrophysiology

Electrophysiological recordings were performed on hippocampal CA3‐CA1 synapses. Field recordings were performed and analyzed as previously described (Gulisano et al., 2019) to assess basal synaptic transmission (BST), LTP, paired‐pulse facilitation (PPF), and post‐tetanic potentiation (PTP). Patch‐clamp recordings were performed and analyzed as previously described (Aceto et al., 2022; Leggio et al., 2019), with minor modifications, to study excitatory post‐synaptic currents (EPSC), AMPA/NMDA ratio, and miniature EPSCs (mEPSCs). See Supplementary Methods for a detailed description.

### 2.4 Electron microscopy

#### 2.4.1 Immunoperoxidase and pre‐embedding procedures

Mice were deeply anesthetized and perfused with saline and a fixative mixture. Brains were post‐fixed, cut into 50 μm parasagittal sections, and processed for pre‐embedding studies. Sections were incubated with anti‐D3 primary antibodies (1:100; ADR‐003, RRID:AB_2039830; Alomone) (Castro‐Hernández et al., 2015; Figure S1) and secondary biotinylated antibodies, followed by avidin‐biotin peroxidase complex and 3,3'diaminobenzidine tetrahydrochloride (DAB). Immunostained sections were then post‐fixed, dehydrated, embedded, and cut in ultrathin sections (~60 nm) for electron microscopy (Melone et al., 2019). Data were collected from CA1 stratum radiatum, and D3 immunoreactive profiles were studied. Microscopical fields were acquired and analyzed to obtain quantitative data on the density of D3 positive profiles and their subcellular distribution at asymmetric synapses. See Supplementary Methods—Data S1 for a detailed description.

#### 2.4.2 Electron microscopy of hippocampal slices

Hippocampal slices were fixed right after electrophysiological recordings, embedded, and sectioned according to the same protocol applied for pre‐embedding studies (Gulisano et al., 2019; Melone et al., 2019). Synaptic domains were identified based on established criteria, and quantitative analysis of vesicle pool, number of docked vesicles, area of spines, length of the post‐synaptic density (PSD), and the proportion of perforated synapses was carried out. See Supplementary Methods—Data S1 for a detailed description.

### 2.5 Western blot on hippocampal slices

Hippocampal slices (*n* = 3 from *n* = 4 mice/group) were homogenized in lysis buffer (62.5 mM Tris–HCl pH 6.8, 3% SDS) in the presence of phosphatase (Phosphatase Inhibitor Cocktail, Calbiochem) and protease inhibitors (Protease Inhibitor cocktail, Sigma), and sonicated three times for 1 min (Acquarone et al., 2019; Gulisano et al., 2019). Protein concentrations were determined by Pierce BCA protein assay kit (Thermo Fisher Scientific) and equal amounts of proteins (30 μg) were diluted in Laemmli buffer, boiled, and resolved by SDS‐PAGE. The following primary antibodies were incubated overnight at 4°C all diluted 1:1000: rabbit anti‐PSD‐95 (Abcam #18258), mouse anti‐pGluA1Ser845 (Cell Signaling #8084), mouse antiGLuA1 (Millipore #2263), rabbit anti‐pCREBS133 (Millipore #06–519), mouse anti CREB (Thermo Fisher Scientific #MA1‐083), and mouse anti‐GAPDH (Abcam #9484). ECL signals were acquired and analyzed with the UVITEC imaging system (Cambridge Alliance). Densitometric analysis was performed with UVItec software after normalization with loading controls. Results are expressed as fold change versus vehicle‐treated WT slices, which were considered equal to 1. Molecular weights for immunoblot analysis were determined using Precision Plus proteinTM standards (Biorad).

### 2.6 Behavioral studies

Open Field, Novel Object Recognition (NOR) and Location (NOL) (Tropea et al., 2021; Tropea, Sanfilippo, et al., 2022), and Morris Water Maze (Gulisano et al., 2018) were performed as previously described. For NOR and NOL, mice underwent the training session (T1), and after 24 h, the testing session (T2) to assess memory retention. We used two different protocols varying in the duration of exposure to the two familiar objects during T1: (i) a short protocol (T1 = 3 min), which is normally insufficient to trigger long‐term memory (LTM) formation; (ii) a long protocol (T1 = 10 min) to evaluate LTM.

Stereotaxic surgery for intrahippocampal cannula implantation was performed as described in Tropea, Torrisi, et al., 2022. Customized home‐made cannulas were implanted in dorsal hippocampi under anesthesia, and mice recovered for at least 6–8 days. NGB‐2904 was bilaterally infused 15 min before the T1 phase of NOR. See Supplementary Methods—Data S1 for a detailed description.

### 2.7 Statistics

All experiments were performed in blind with respect to treatment or genotype. Data were expressed as mean ± standard error mean (SEM). The level of significance was set at *p* < 0.05. Statistical analysis was performed by Systat 9, GraphPad Prism Software 9.5.1, and SigmaPlot software 14.0. Based on preliminary analyses of normal distribution by Shapiro–Wilk normality test, we performed: (i) ANOVA for repeated measures to analyze LTP, PTP, PPF, BST, and MWM latency; (ii) one‐way ANOVA with Bonferroni's post‐hoc correction for open field, NOR and NOL in aged mice, MWM probe test; (iii) two‐tailed t‐test when comparing two samples; (iv) one sample *t* test to compare D with zero in NOR and NOL; (v) Kruskal–Wallis One Way Analysis of Variance on Ranks with Student–Newman–Keuls for multiple comparison for western blot analyses. Given the non‐normal distribution of electron microscopical data, as assessed by D'Agostino and Pearson normality test, we used nonparametric contingency analysis with Fisher's test for pre‐embedding electron microscopy data and nonparametric Kruskal–Wallis test and Dunn's multiple comparison test for electron microscopy measures obtained from hippocampal recorded slices.

## 3 RESULTS

### 3.1 D3R blockade or genetic deletion enhances hippocampal plasticity

To examine the impact of D3R blockade on hippocampal synaptic transmission and plasticity we performed electrophysiological studies at CA3‐CA1 synapses. We first investigated the role of D3Rs in the weak form of long‐term potentiation (LTP1), also known as early LTP, a transient modification in synaptic strength evoked by a weak tetanic stimulation (1 TBS) that requires additional reinforcement to be converted into a more persistent and stable LTP2.

**FIGURE 1:**
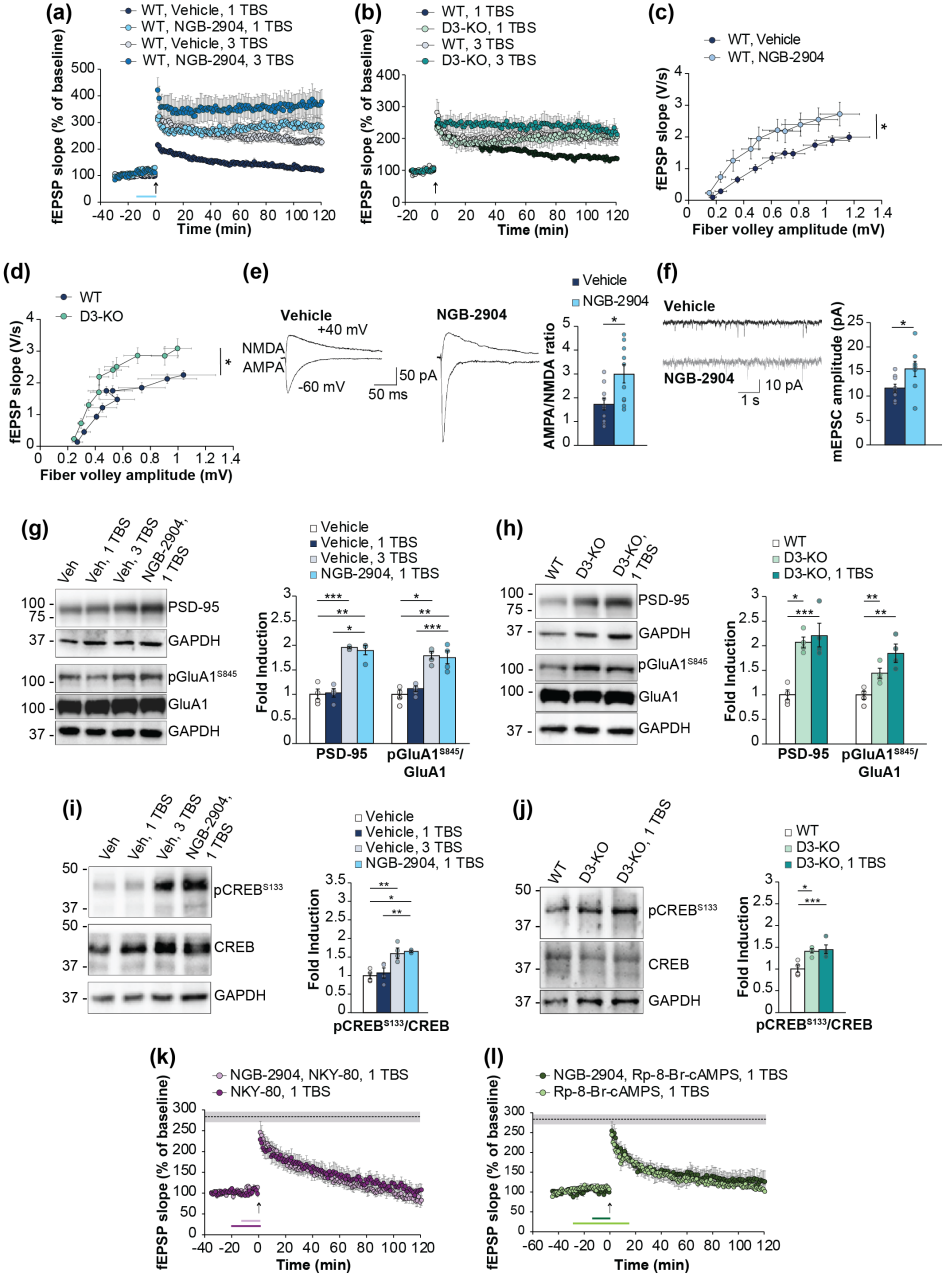
Effects of D3R blockade on hippocampal synaptic function. (a) NGB‐2904 (1 μM for 15 min before a weak tetanic stimulation, 1 TBS) converted LTP1 into LTP2 (F_(1,12)_ = 75.341, *p* < 0.0001, *n* = 7/7) and enhanced potentiation induced by a strong tetanic stimulation (3 TBS) (F_(1,13)_ = 4.745, *p* = 0.048, *n* = 7). (b) D3‐KO slices developed LTP2 after 1 TBS (F_(1,11)_ = 10.194, *p* = 0.009, *n* = 7/7). No differences were found in 3 TBS‐induced LTP2 in D3‐KO vs. WT slices (F_(1,9)_ = 1.507, *p* = 0.251, *n* = 6/6). (c) fEPSP slope measured during BST increased in NGB‐2904‐treated slices compared with vehicle (*n* = 10/13; F_(1,21)_ = 4.416, *p* = 0.048 vs. vehicle), whereas no difference was found in fiber volley amplitude (F_(1,21)_ = 0.304, *p* = 0.587). (d) fEPSP slope measured during BST increased in slices from D3‐KO mice compared with WT (*n* = 10/12; F_(1,20)_ = 5.020, *p* = 0.037), whereas no difference was found in fiber volley amplitude (F_(1,20)_ = 0.049, *p* = 0.828). (e) Representative traces of AMPA‐ and NMDA‐receptor‐mediated EPSCs and bar graph of AMPA/NMDA ratio after 10 min incubation with vehicle or NGB‐2904 (1 μM) (t_(18)_ = 2.774; *p* = 0.012; *n* = 10/10). (f) Representative traces and graph showing that the peak amplitude of mEPSCs increased in slices incubated with NGB‐2904 (t_(15)_= 2.333, *p* = 0.034). (g) Representative WB images of PSD‐95, GluA1, and pGluA1 (cropped images based on MW) performed in lysates from slices treated for electrophysiology stored at 1 min after tetanus (*n* = 3 slices/lane). GAPDH was used as loading control. On the right, bar graph showing the increase of PSD‐95 (*p* = 0.046) and pGluA1 (*p* < 0.001) expression in WT slices treated with NGB‐2904 + 1 TBS. (h) PSD‐95 (*p* = 0.049) and pGluA1 expression (*p* = 0.009) increased in D3‐KO slices in basal conditions and after 1 TBS (*p* < 0.001; *p* = 0.009). (i) pCREB expression increased in WT slices treated with NGB‐2904 + 1 TBS (*p* = 0.005) and (j) D3‐KO slices in basal conditions (*p* = 0.038) and after 1 TBS (*p* < 0.001). (k) NKY‐80 (10 μM, 20 min before tetanus) prevented the NGB‐2904‐induced LTP2 (F_(1,12)_ = 84.83, *p* < 0.0001, *n* = 7). Shaded area with dotted line corresponds to mean ± SEM of last point of potentiation induced by NGB‐2904 + 1 TBS shown in Figure 1a. (l) Rp‐8Br‐cAMPs (20 μM, 30 min before and 15 min after tetanus) prevented the NGB‐2904‐induced LTP2 (F_(1,12)_ = 52.806, *p* < 0.0001 vs. NGB‐2904 + 1 TBS, *n* = 7). Data expressed as mean ± SEM. *p < 0.05, **p < 0.01, ***p < 0.001. [Correction added on 4th October after first online publication: Figure 1 has been replaced as it had some material and data copying errors.]

Experiments of field recordings confirmed that 1 TBS induced LTP1 in vehicle‐treated slices from adult WT animals (Gulisano et al., 2019), whereas a treatment with the D3R antagonist NGB‐2904 for 15 min before 1 TBS was sufficient to elicit LTP2 comparable to that obtained by a strong tetanic stimulation (3 TBS) (Figure 1a). Blocking D3Rs with NGB‐2904 further enhanced 3TBS‐induced potentiation (Figure 1a).

To provide genetic evidence of the involvement of D3Rs in synaptic transmission and plasticity, we performed a series of additional experiments by using D3 knock out mice (D3‐KO) and found that D3‐KO slices were capable to develop a LTP2 after 1 TBS (Figure 1b), whereas 3 TBS‐induced LTP2 was comparable to that evoked in WT slices (Figure 1b).

Because LTP involves both pre‐ and post‐synaptic modifications, we examined pre‐synaptic forms of plasticity while blocking LTP inductive mechanisms with the NMDA antagonist (2R)‐amino‐5‐phosphonovaleric acid (APV). We observed that NGB‐2904 did not alter the presynaptic component of post‐tetanic potentiation (PTP; Figure S2a) or paired‐pulse facilitation (PPF; Figure S2b), suggesting that presynaptic mechanisms were not involved.

We then analyzed basal synaptic transmission (BST) and found that blockade of D3Rs increased fEPSP slopes obtained during input–output recordings in NGB‐2904‐treated compared to vehicle‐treated slices (Figure 1c, Figure S2c) and in D3‐KO compared to WT slices (Figure 1d, Figure S2e). To better understand whether the BST enhancement was due to pre‐ and/or post‐synaptic modifications we analyzed afferent volley amplitude, an index of pre‐synaptic firing, that was unchanged, confirming that D3R blockade mainly acted via a post‐synaptic mechanism (Figure 1c,d, Figure S2d,f).

As a further insight, we conducted electrophysiological experiments using whole‐cell patch‐clamp recordings in hippocampal slices (Ripoli et al., 2014). We studied the input–output properties of AMPAR‐ and NMDAR‐mediated excitatory post‐synaptic currents EPSCs in response to fixed‐intensity test stimuli. Analysis of the AMPA/NMDA ratio indicated an increase of the AMPAR‐mediated synaptic responses following treatment with the D3R antagonist NGB‐2904 (Figure 1e), thus suggesting an enhancement of synaptic strength.

This effect was further confirmed by assessing miniature excitatory post‐synaptic currents (mEPSCs) that exhibited an increase in mEPSC amplitude after the extracellular administration of NGB‐2904 (Figure 1f, Figure S2g). No differences were observed in mEPSC frequency (Figure S2h,k), rise time (Figure S2i,l), and decay time (Figure S2j,m), confirming that D3R blockade primarily induced post‐synaptic modifications.

### 3.2 D3R blockade induced modifications in post‐synaptic protein expression

Since electrophysiological data indicated a primary involvement of post‐synaptic D3Rs, we investigated the effect of D3R blockade on post‐synaptic machinery. We therefore assessed post‐synaptic protein expression performing western blot (WB) experiments on the same hippocampal slices used for LTP electrophysiological recordings (Gulisano et al., 2019). We first evaluated the levels of PSD‐95, whose expression strongly correlates with synaptic strengthening, and observed a significant increase in its expression in slices treated with NGB‐2904 paired with 1 TBS (+89%; Figure 1g), comparable to that found in vehicle‐treated slices that underwent a strong tetanization (+96%; Figure 1g). The increase of PSD‐95 expression was also evident in D3‐KO slices that received 1 TBS (+121%; Figure 1h) and 3 TBS (+98%; Figure S3a). Interestingly, a similar increase was present in D3‐KO slices that did not receive tetanic stimulation (+113%; Figure 1h).

Since PSD‐95 modulates the trafficking and localization of glutamate receptors, we examined the expression of AMPA receptor subunit GluA1 phosphorylated at Ser845 (pGluA1), known to influence synaptic strength and plasticity. In hippocampal slices treated with NGB‐2904 and 1 TBS, the expression of pGluA1 increased and was comparable to that found in vehicle‐treated slices that underwent a strong tetanization (+75% and + 79%, respectively; Figure 1g). We observed the same increase in D3‐KO slices after 1 TBS (+76%; Figure 1h), 3 TBS (+68%; Figure S3b), but also in basal conditions (+44%; Figure 1h).

Since D3Rs are known to be negatively coupled to AC (Robinson & Caron, 1997), their blockade was expected to increase the cAMP/PKA signaling pathway, leading to CREB phosphorylation, which is associated with LTP2 and memory formation (Teich et al., 2015). Consistently, CREB phosphorylated at Ser133 (p‐CREB) increased in slices treated with NGB‐2904 and 1 TBS (+66%; Figure 1i), as well as in D3‐KO slices treated with either 1 TBS, 3 TBS, or vehicle alone (+45%, +61%, +39%; Figure 1j, Figure S3b).

To further investigate the influence of D3Rs on the cAMP/PKA/CREB intracellular signaling pathway, we performed a series of LTP recordings in slices from WT animals treated with NGB‐2904 paired with inhibitors of the cAMP pathway. Our experiments demonstrated that both the inhibition of adenylyl cyclase by NKY‐80 (Figure 1k) and the inhibition of PKA by Rp‐8‐Br‐cAMPS (Figure 1l) prevented NGB‐2904 from converting LTP1 into LTP2, thus confirming that the LTP enhancement induced by D3R blockade was cAMP/PKA dependent. Control experiments showed that NKY‐80 or Rp‐8‐Br‐cAMPS did not affect LTP evoked by 1 TBS (Figure 1k,l).

### 3.3 D3Rs ultrastructural localization in hippocampal CA1 area

**FIGURE 2:**
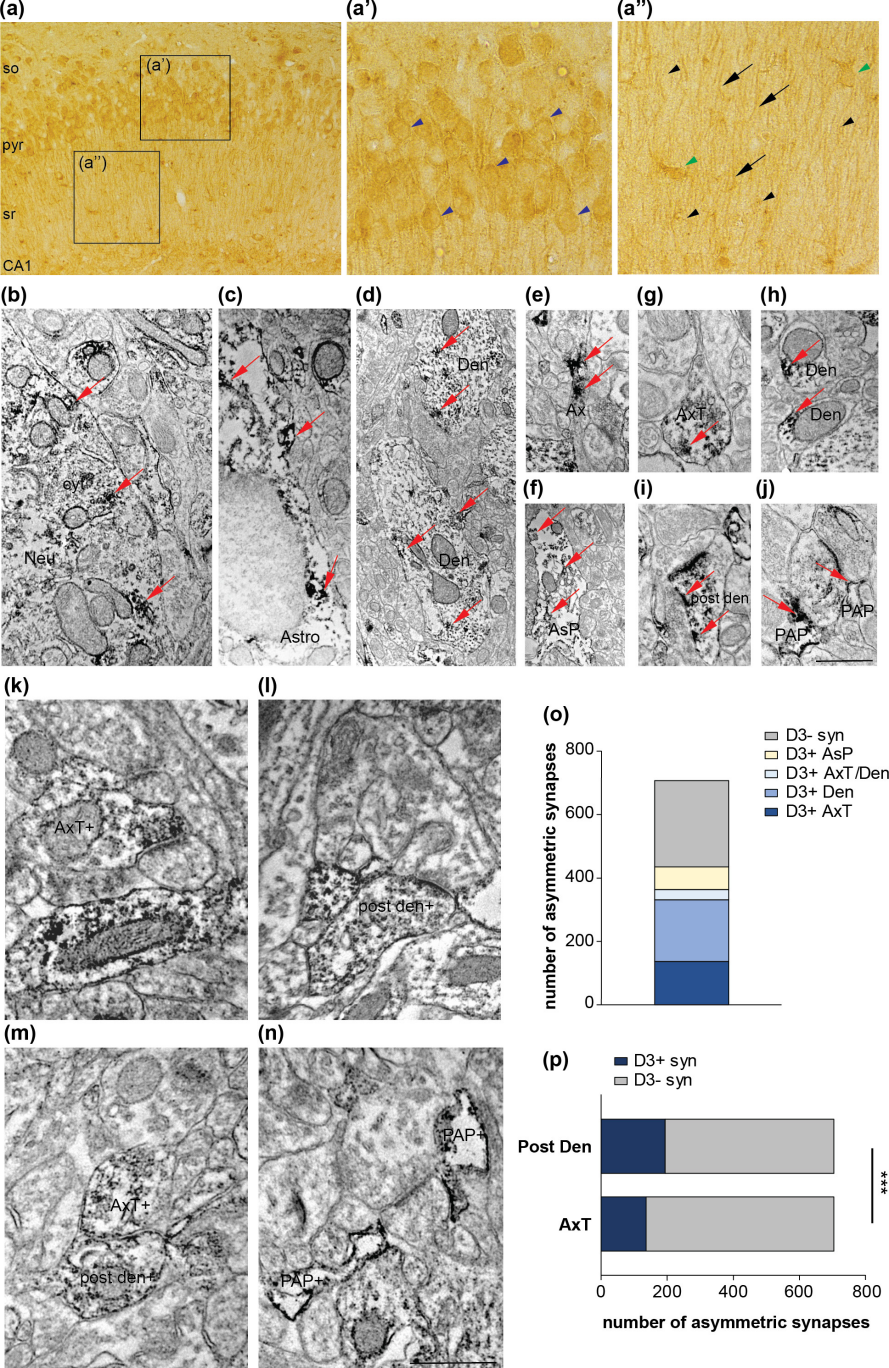
Cellular and subcellular localization of D3Rs in CA1 adult hippocampus. (a) Light microscopy of D3R immunoreactivity (IR) in stratum oriens (so), pyramidalis (pyr), and radiatum (sr) unveiled neuronal‐ and glial‐like positive cells (blu arrowheads in a’ and green arrowheads in a”, respectively), positive profiles [arrows in (a”)], and small positive (+) puncta [black arrowheads in (a”)]. (b–j) Pre‐embedding electron microscopy of pyr‐sr layers identified immuno‐positive electron dense products (examples of them marked with red arrows in all panels) in the cytoplasm (cyt) of neurons [(Neu in (b)], astrocytes [Astro in (c)], dendrites [Den in (d)], axons [Ax in (e)], proximal glial processes [AsP in (f)], axon terminals [AxT in (g)], small distal dendrites [den in (h)], post‐synaptic dendrites [post den in (i)], and peri‐synaptic astrocytic processes [PAP in (j)] in close relationship with synaptic elements. (k–n) Pre‐embedding microscopy showed that at asymmetric synapses, D3Rs localize at axon terminals [AxT+ in (k)], post‐synaptic dendrites [post den+ in (l)], at both AxT and post den (m), and in pre‐synaptic astrocytic processes [PAP+ in (n)] in close relationship with synaptic domains. (o) Graph illustrating the proportion between positive synaptic elements and negative synapses. Out of 704 asymmetric synapses, 137 (19.5%) were positive at AxT (D3+ AxT), 194 (27.6%) at post‐synaptic dendrites (D3+ Den), 33 (4.7%) at both AxT and post‐synaptic dendrites (D3+ AxT/Den), 68 (9.6%) at peri‐synaptic astrocytic processes (D3+ AsP), and 272 (38.6%) were negative (D3− syn). (p) Comparison between pre‐synaptic (AxT) and post‐synaptic domains (Post Den) showed that in D3 positive synapses (D3+ syn), D3Rs were mainly expressed in the post‐synaptic dendrites (19.5% vs. 27.6%, *p* = 0.0004, Fisher's exact test). Scale Bar: 200 μm for (a), 120 μm for (a’)–(a”), 500 nm for (b)–(n). ***p ≤ 0.001.

Prompted by the pronounced effects of D3R antagonism on hippocampal synaptic plasticity, we evaluated the localization of D3Rs in the hippocampus, focusing on the CA1 area, where electrophysiological recordings were performed. Light microscopy of intact adult CA1 revealed diffuse D3R immunoreactivity (IR) in *stratum oriens* (so), *pyramidalis* (pyr), and *radiatum* (sr) characterized by neuronal‐ and glial‐like positive cells, positive profiles, and small positive (+) puncta dispersed in the neuropil (Figure 2a,a″).

Pre‐embedding electron microscopy of pyr‐sr layers confirmed the presence of immuno‐positive products in the cytoplasm of neurons and astrocytes, deciphered the dendritic, axonal, and glial nature of D3R+ profiles, and unveiled the expression of D3Rs in axon terminals, small distal dendrites, post‐synaptic dendrites, and distal astrocytic processes in close relationship with synaptic elements (Figure 2b–j).

We next estimated quantitatively the degree of expression of D3Rs in synaptic domains of asymmetric synapses in sr. D3Rs were detectable in axon terminals (Figure 2k,o,p), post‐synaptic dendrites (Figure 2l,o,p), in both AxT and post‐synaptic dendrites (Figure 2m,o), and in distal astrocytic processes (Figure 2n,o) in close proximity to synaptic domains. Analysis of D3R distribution between synaptic domains of positive asymmetric synapses revealed that D3R expression was higher in the post‐synaptic dendrites than in axon terminals (Figure 2p).

### 3.4 D3R blockade or genetic deletion induce long‐term memory formation

Our results indicated that D3R blockade induces the synaptic changes underlying LTP and CREB phosphorylation, intracellular signaling events that play an essential role in the molecular mechanisms underlying memory formation. Thus, we examined whether blocking D3Rs exerted a cognitive‐enhancing effect. We used modified protocols of NOR and NOL tasks with a short training phase (T1) that, normally, is sufficient to induce short‐term memory lasting up to 6–8 h (Blokland & Sesia, 2023) but does not allow to discriminate between the familiar and the novel object after a 24‐h interval testing phase (Bollen et al., 2014; Gulisano et al., 2019; Palmeri et al., 2017).

**FIGURE 3:**
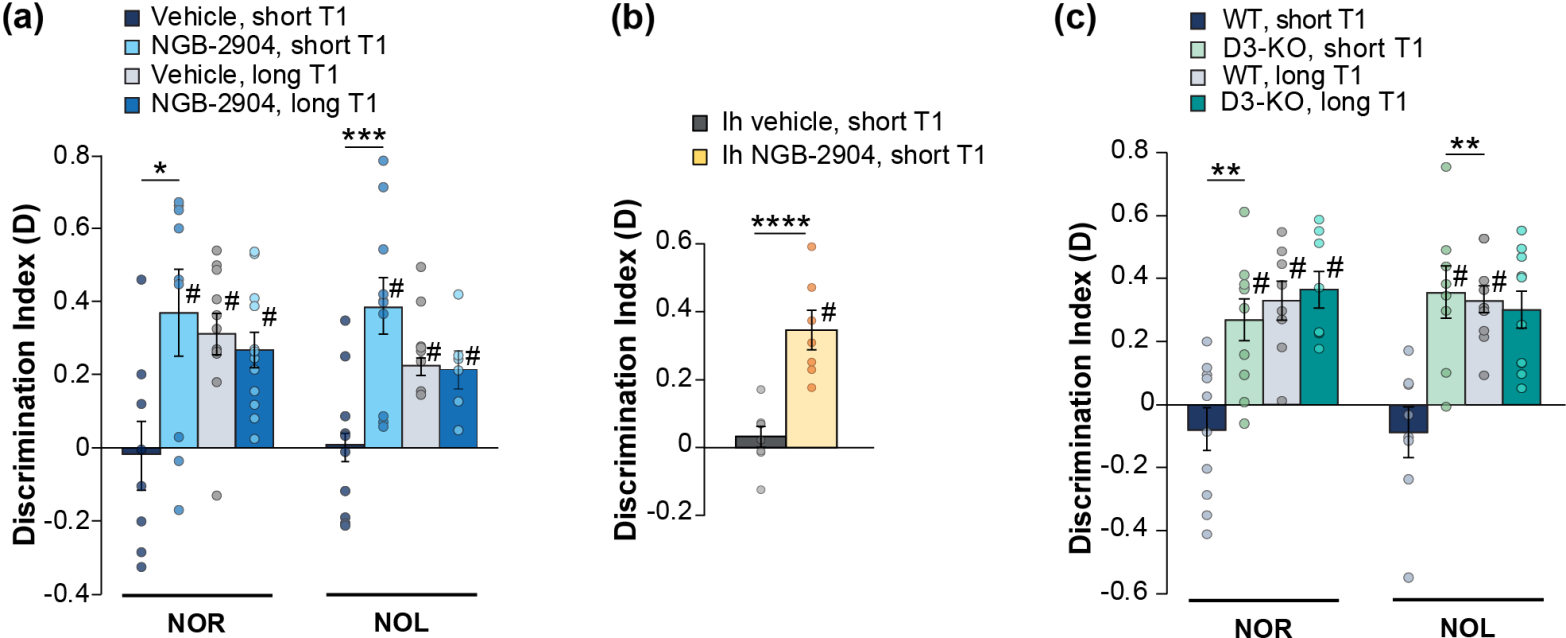
D3R blockade or genetic deletion stimulates long‐term memory formation. (a) A short training (3 min in T1) in NOR and NOL did not allow long‐term memory (LTM) formation in vehicle‐treated mice (discrimination index *D* = 0 NOR: *p* = 0.869, *n* = 8; NOL: *p* = 0.989, *n* = 10) bur was sufficient for LTM formation in NGB‐2904‐treated WT animals (D ≠ 0 NOR: *p* = 0.011, *n* = 9; NOL: *p* = 0.001, *n* = 10). Mice treated with vehicle or NGB‐2904 formed LTM if exposed to a long T1 (10 min) (D ≠ 0 NOR: *p* < 0.0001, *n* = 11; NGB‐2904 *p* < 0.0001, *n* = 12; NOL: Vehicle *p* < 0.0001, *n* = 8; NGB‐2904 *p* = 0.009, *n* = 6). Planned comparison confirmed the difference between vehicle‐ and NGB‐2904‐treated mice that underwent a short T1 in NOR (F_(3,36)_ = 4.364), *p* = 0.01; Bonferroni's *p* = 0.012) and NOL (F_(3,30)_ = 6.977, *p* = 0.001; Bonferroni's *p* = 0.001). (b) Intrahippocampal injections (Ih) of NGB‐2904 induced LTM after a short T1 (D ≠ 0: *p* = 0.001, n = 7). Two‐group comparison confirmed that treatment affected D (t_(13)_ = 5.107, *p* < 0.0001). (c) D3‐KO mice that underwent a short T1 formed LTM in NOR (D ≠ 0; *p* = 0.003, *n* = 10) and NOL (*p* = 0.004, *n* = 8). A long T1 evoked LTM in both WT and D3‐KO mice (D ≠ 0 in NOR: WT *p* = 0.001, *n* = 8; D3‐KO *p* < 0.0001, *n* = 8; NOL: WT *p* < 0.0001, *n* = 9; D3‐KO *p* = 0.001, *n* = 10). Planned comparison confirmed the difference between WT and D3‐KO mice that underwent a short T1 in NOR (F_(3,32)_ = 10.163, *p* < 0.0001; Bonferroni's *p* = 0.002) and NOL (F_(3,31)_ = 9.077, *p* < 0.0001; Bonferroni's *p* = 0.001).Data expressed as mean ± SEM. *p < 0.05; **p ≤ 0.01; ***p ≤ 0.001; ****p ≤ 0.0001; # difference from 0.

Vehicle‐treated mice did not discriminate between objects or their positions after a 24‐h interval (Figure 3a), whereas mice treated with intraperitoneal (ip) administration of NGB‐2904 showed a high discrimination index (Figure 3a). We then evaluated the effect of D3R blockade on LTM. To this end, mice were trained for a longer time in T1 (10 min) that ensures memory formation after 24 h. Under these conditions, vehicle‐treated and NGB‐2904‐treated animals exhibited similar discrimination indices (Figure 3a) in both the NOR and NOL tests.

Considering that systemic ip treatment with NGB‐2904 can block D3Rs in various brain areas, we investigated the role of hippocampal D3Rs in memory formation by administering NGB‐2904 via intrahippocampal injections. Our findings revealed that mice treated with NGB‐2904 (injected 15 min before the short T1) spent a longer time exploring the novel object in T2 compared to vehicle‐treated mice (Figure 3b), confirming the cognitive‐enhancing effect of hippocampal D3Rs blockade.

The same results were obtained in D3‐KO mice that were capable to form memory when tested with a NOR and a NOL task after a short T1 (Figure 3c), whereas presented normal recognition and spatial memory after a long T1 (Figure 3c).

Total exploration time was not affected by ip or intrahippocampal treatment with NGB‐2904 (Figure S4a–c) or D3‐R genetic deletion (Figure S4d,e). Furthermore, treatment and genotype did not modify the Open Field results (Figure S4f,g), suggesting that mouse exploratory behavior did not influence memory performance.

No significant differences were detected when analyzing data obtained with NOR, NOL and OF tests in males versus females (Figure S5).

### 3.5 D3R levels and D3R expression at excitatory synapses are age‐dependent

Our next goal was to investigate the role of D3Rs in aging and whether the positive effect of D3R blockade on synaptic plasticity and memory could be exploited against the age‐related cognitive impairment. To accomplish this, we utilized animal models of physiological aging, specifically WT mice aged 18–22‐month‐old, previously shown to exhibit a deficit of LTP and memory (Palmeri et al., 2013).

**FIGURE 4:**
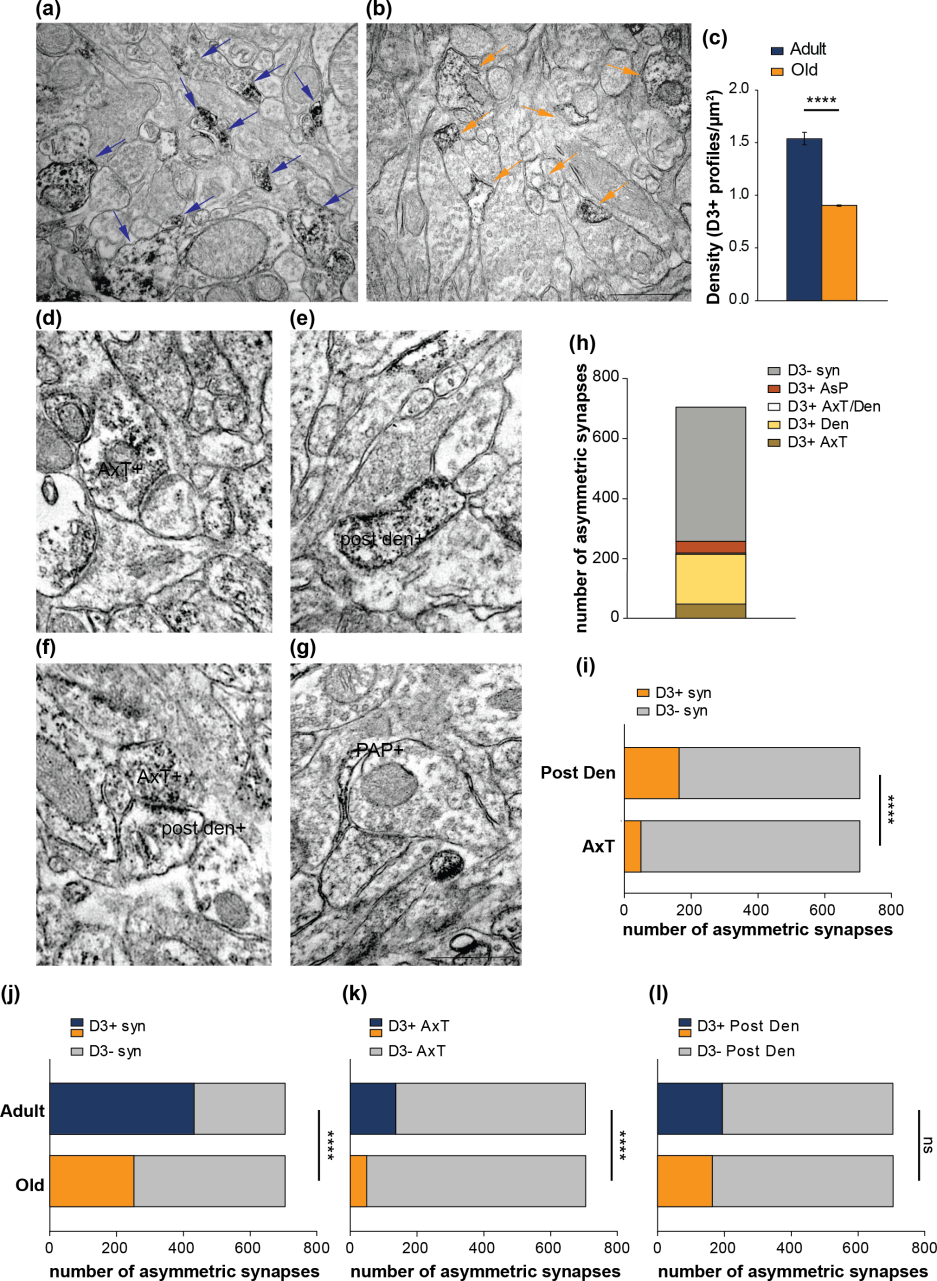
Modifications of D3Rs expression and distribution in hippocampi from aged mice. (a, b) Representative examples of electron microscopical fields immunoreacted for D3R displaying several positive profiles (with dark electrodense products) marked arrows from adult (blu arrows) and aged (orange arrows) mice. (c) Graph reporting the comparison between the density of D3+ profiles/μm^2^ of adult (1.5 ± 0.05 profiles/μm^2^; from 167 microscopical fields/3 mice) and aged mice (0.9 ± 0.03 profiles/μm^2^; from 173 microscopical fields/3 mice. (d–g) Pre‐embedding microscopy showed that D3Rs are expressed in axon terminals [AxT+ in (d)], post‐synaptic dendrites [post den+ in (e)], at both AxT and post den (in f), and in distal astrocytic processes [PAP+ in (g)] touching synaptic domains. (h) Graph illustrating the proportion between positive synaptic elements and negative synapses [out of 708 asymmetric synapses, 48 (6.8%) were positive at AxT, 166 (23.4%) at post den, 4 (0.6%) at both AxT and post den, 39 (5.5%) at peri‐synaptic astrocytic processes (AsP), and 451 (63.7%) were negative]. (i) Comparison between pre‐synaptic (AxT) and post‐synaptic (post den) domains showed that in D3+ synapses of aged mice, D3R localization was predominantly post‐synaptic (6.8% vs. 23.4%, *p* < 0.0001, Fisher's exact test). (j–l) Graphs representing the comparison between adult and aged hippocampal synapses indicated a general reduction of D3+ synapses [(j) 61.4% vs 36.3%, *p* < 0.0001, Fisher's exact test)] and that the level of pre‐synaptic expression of D3R was significantly lower in aged mice [(k), 19.5% vs 6.8%, *p* < 0.0001, Fisher's exact test]. Conversely, the degree of post‐synaptic expression was quite comparable between adult and aged mice [(l), 27.6% vs. 23.4%, *p* = 0.077, Fisher's exact test). Scale bar: 500 nm. ****p ≤ 0.0001.

We first studied whether aging modified the expression and localization of D3Rs at the hippocampal level. Quantitative ultrastructural analysis of D3 IR of sr from adult and aged mice revealed that D3+ profiles in aged mice were reduced by ~41% (Figure 4a–c).

In line with results obtained in adult mice (see Figure 2), at excitatory synapses, D3Rs were localized at axon terminals, post‐synaptic dendrites, both axon terminals and post‐synaptic dendrites, and peri‐synaptic astrocytic processes in close relationship with synaptic domains (Figure 4d–h). As in adult mice, quantitative analysis revealed that in aged mice the expression of D3Rs was higher in the post‐synaptic dendrites than in axon terminals (Figure 4i). The comparison of D3R expression at asymmetric synapses between adult and aged mice revealed a reduction of positive synapses in aged mice (Figure 4j). Interestingly, the lowered expression of D3Rs in aged synapses was caused by the reduction of D3Rs at pre‐synaptic sites but not at post‐synaptic dendrites (Figure 4k,l).

### 3.6 D3R blockade or genetic deletion rescues the age‐related synaptic and memory impairment

**FIGURE 5:**
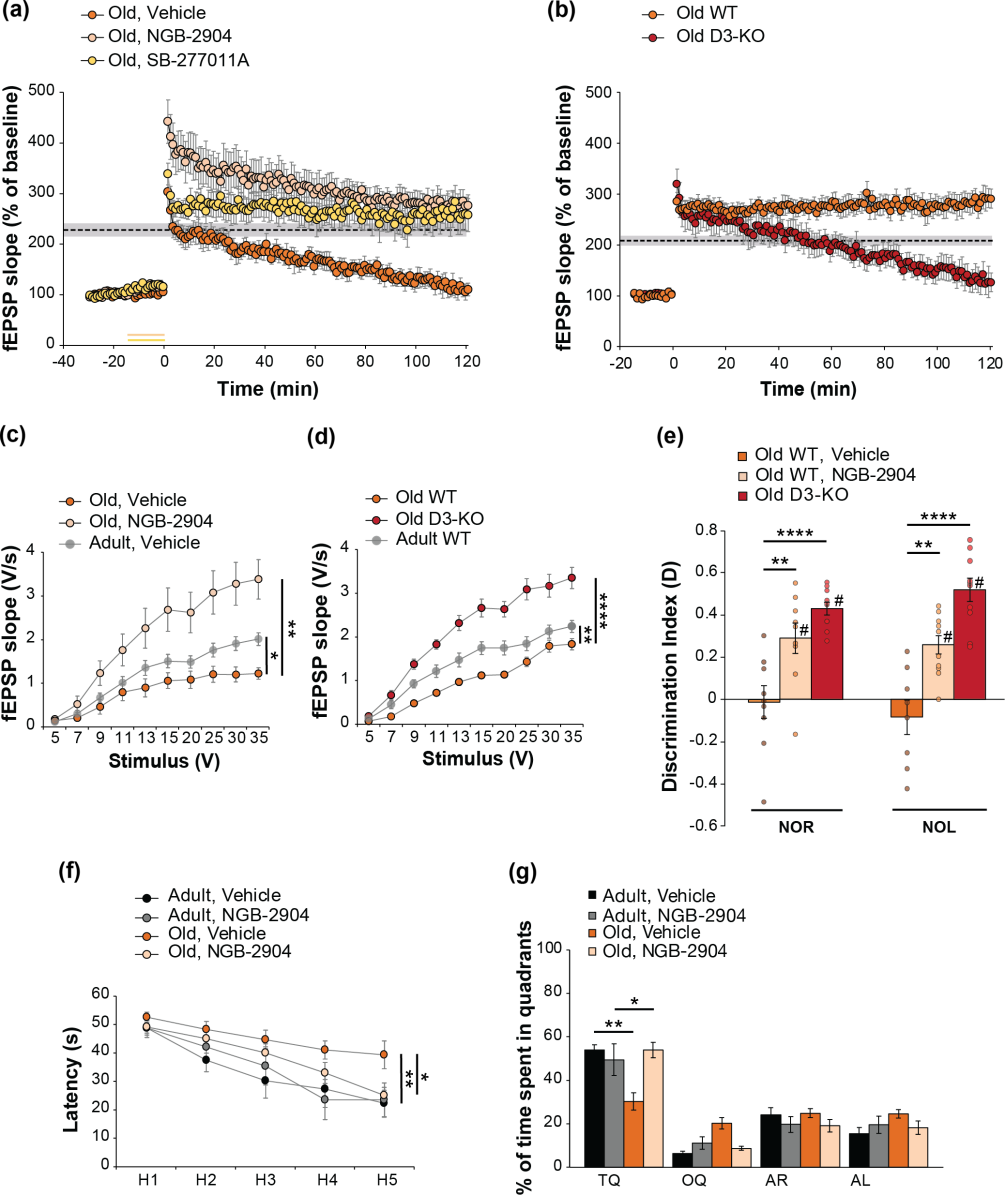
The blockade or genetic deletion of D3Rs rescues the age‐related synaptic and memory impairment. (a) The LTP impairment in slices from old wild type mice (*n* = 6) was restored by the D3‐antagonists NGB‐2904 or SB‐277011A (*n* = 8/6; F_(1,12)_= 17.878, *p* = 0.001; F_(1,10)_ = 10.836, *p* = 0.008). Shaded area with dashed line = mean ± SEM of the last LTP recording in vehicle +3 TBS shown in Figure 1a. (b) Slices from aged D3‐KO mice did not exhibit LTP impairment (*n* = 8/9, F_(1,15)_ = 11.196, *p* = 0.004). Shaded area with dashed line = mean ± SEM of the last LTP recording in WT + 3 TBS shown in Figure 1b. (c) The age‐related impairment of BST (F_(1,21)_ = 5.01, *p* = 0.036) was rescued by NGB‐2904 (F_(1,18)_ = 11.438, *p* = 0.003, *n* = 10/10). (d) Slices from D3‐KO did not present the impairment of BST (F_(1,19)_ = 51.531, *p* < 0.0001, *n* = 11/10). (e) The age‐related impairment of memory (D = 0 NOR: *p* = 0.981, *n* = 10; NOL: *p* = 0.341; *n* = 8) was rescued by NGB‐2904 (D ≠ 0 NOR: *p* = 0.004, *n* = 9; NOL: *p* < 0.0001, *n* = 10) or in D3‐KO mice (NOR: *p* < 0.0001, *n* = 10; NOL: *p* < 0.0001, *n* = 10). Planned comparisons confirmed the rescuing effect of NGB‐2904 (Bonferroni's *p* < 0.01) and D3R deletion (*p* < 0.0001). (f) The age‐related decrease of latency (F_(1,17)_ = 7.983, *p* = 0.012, *n* = 10/9) was rescued by NGB‐2904 (F_(1,19)_ = 4.792, p = 0.041, *n* = 11). (g) Aged mice treated with NGB‐2904 spent more time in TQ (*p* < 0.0001). Planned comparisons confirmed that the age‐related memory impairment was rescued by NGB‐2904 (*p* = 0.001). Data expressed as mean ± SEM. *p < 0.05, **p < 0.01, ****p < 0.0001; # difference from 0. AR, adjacent right; AL, adjacent left; OQ, opposite quadrant; TQ, target quadrant.

To assess whether D3R blockade might rescue the age‐related cognitive phenotype, we first studied LTP in hippocampal slices and found that a perfusion with the D3R antagonist NGB‐2904 restored the age‐related impairment of potentiation (Figure 5a). To further confirm the positive effect of D3R blockade, we used another D3R antagonist, SB‐277011A, which also normalized LTP in slices from aged WT mice (Figure 5a). We then evaluated LTP in hippocampal slices from aged D3‐KO animals and found that they did not exhibit the expected age‐related impairment in LTP but a normal potentiation after a strong tetanus (Figure 5b). Because D3R blockade or genetic deletion increased BST in slices from adult animals (see Figure 1c,d), we also studied whether acting on these receptors could exert a positive effect on BST in old animals. A pharmacological blockade with NGB‐2904 (Figure 5c) or the genetic deletion in D3‐KO models (Figure 5d) not only restored the age‐related impairment of BST but improved fEPSP responses when compared with slices from adult animals, without changing afferent volley (Figure S6a,b).

Thus, D3R pharmacological blockade or genetic deletion had a protective effect against the age‐dependent impairment of hippocampal synaptic transmission and plasticity.

Next, we performed the behavioral test of NOR and NOL on aged WT mice treated with ip injections of the D3R antagonist NGB‐2904 and on D3‐KO animals to understand whether D3R blockade or genetic deletion could rescue the age‐related memory impairment. NGB‐2904‐treated and D3‐KO aged mice both presented normal recognition memory in a NOR test, as they spent more time in exploring the novel compared with the familiar object resulting in a restored D index (Figure 5e). We obtained the same results in a NOL test where aged mice treated with NGB‐2904 and aged D3‐KO presented a normal D index due to the higher time of exploration for the object located in a novel position compared to the old one (Figure 5e). Total exploration time was mot modified by treatment or genotype in NOR and NOL (Figure S6c). The Open field test confirmed that exploratory behavior was unaffected in old WT treated with NGB‐2904 and D3‐KO animals (Figure S6d,e).

Consistent with findings in adult mice, we observed no sex differences in aged WT mice, animals treated with NGB‐2904, or in D3‐KO models (Figure S7).

The effect induced by D3R blockade was also verified with the Morris Water Maze test, a behavioral test widely used to assess spatial learning and reference memory in rodents. Aged mice showed an impairment of spatial memory as they need a higher time (latency) to reach the hidden platform during the spatial learning test compared to adult controls (Figure 5f), but a treatment with NGB‐2904 was capable to rescue this deficit (Figure 5f). Then, we assessed reference memory with the probe test that evaluated the amount of time spent in each quadrant of the maze after removing the platform. For each experimental group, we first compared the time spent in the TQ, where the platform was located during training, with other quadrants to verify whether mice retained reference memory. We found that adult mice treated with vehicle or NGB‐2904 spent significantly more time in the TQ compared with other quadrants (Figure 5g). Conversely, aged mice treated with vehicle spent a similar amount of time in the four quadrants indicating an impairment of reference memory that was rescued after treatment with NGB‐2904 (Figure 5g). Planned comparison indicated that adult mice treated with vehicle or NGB‐2904 and aged mice treated with NGB‐2904 spent a similar amount of time exploring the TQ, whereas aged mice treated with vehicle spent less time exploring the TQ (Figure 5g). A visible platform trial did not reveal any significant difference in the time to reach the platform among the 4 groups of mice (Figure S6f).

### 3.7 D3R blockade or genetic deletion elicits post‐synaptic modifications underlying the rescue of age‐induced synaptic‐impairment

**FIGURE 6:**
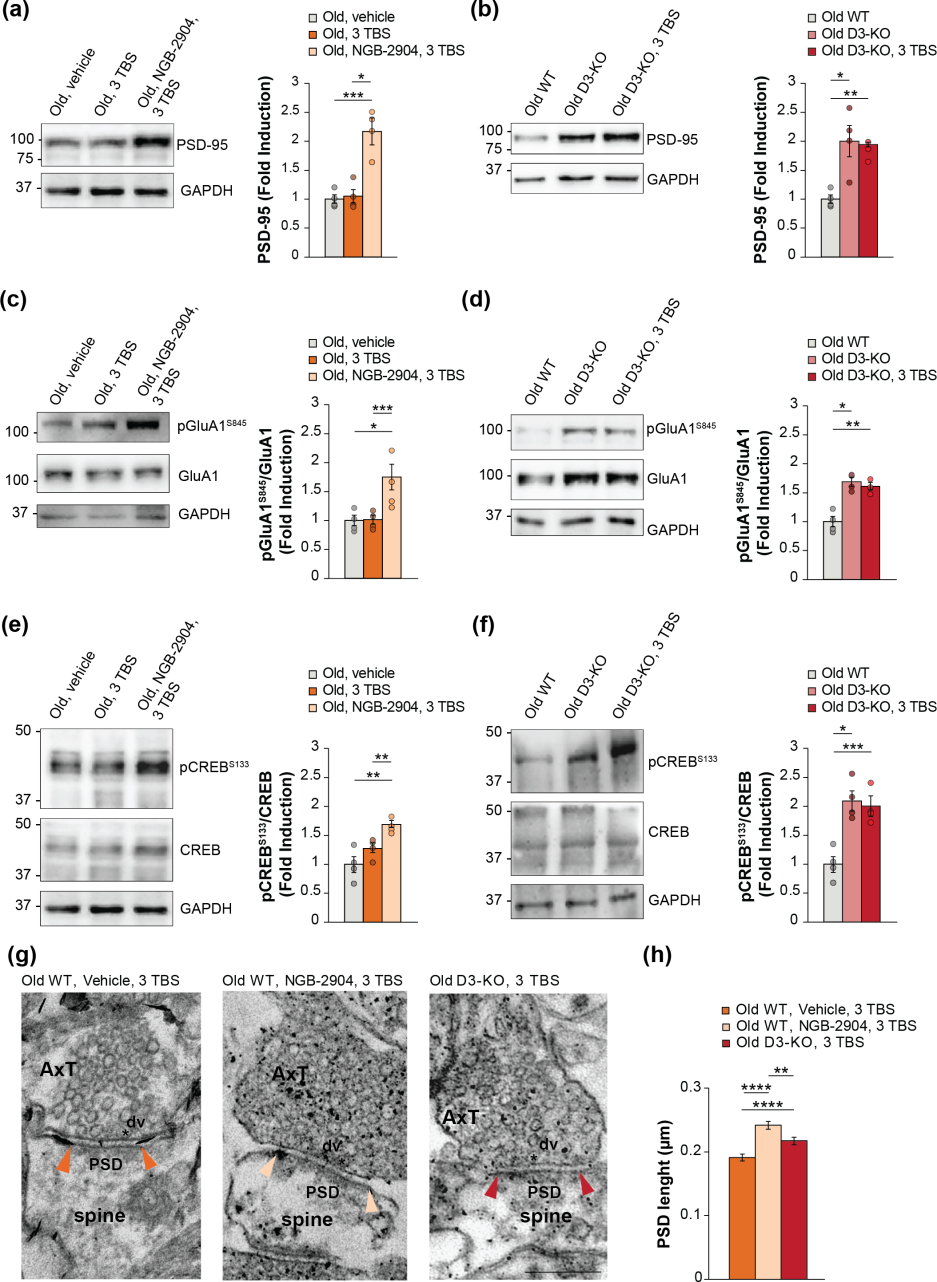
D3R blockade or genetic deletion rescues the age‐related impairment of post‐synaptic proteins and PSD length. (a) Representative images of WB experiments of PSD‐95 (cropped images based on MW) performed on hippocampal slices treated for electrophysiological experiments stored at 1 min after treatment (*n* = 3 slices/lane). Bar graph showing that a treatment with NGB‐2904 rescued PSD‐95 expression in slices from aged WT mice that underwent a strong tetanic stimulation (*p* = 0.049). GAPDH was used as loading control. (b) Slices from aged D3‐KO mice presented an increase of PSD‐95 expression either in basal conditions (*p* = 0.017) or after 3 TBS (p = 0.004) vs. old WT. (c) NGB‐2904 rescued pGluA1 expression in slices from aged WT mice that underwent a strong tetanic stimulation (*p* < 0.001). (d) Slices from aged D3‐KO mice presented an increase of pGluA1 expression either in basal conditions (*p* = 0.029) or after 3 TBS (*p* = 0.001) versus old WT. (e) NGB‐2904 rescued pCREB expression in slices from aged WT mice (*p* = 0.009). The same lane for GAPDH is shown here and in panel (a) (loading control for PSD‐95). (f) Slices from aged D3‐KO mice presented an increase of pCREB expression either in basal conditions (*p* = 0.029) or after 1 TBS (*p* = 0.001) versus old WT. (g) Representative asymmetric axo‐spinous synapses of CA1 stratum radiatum from hippocampal slices from aged mice previously treated for electrophysiological recordings. Colored arrowheads point to PSD edges for each synapse from different experimental conditions. Asterisks mark docked vesicles (dv) at the active zone of the axon terminals (AxT). (h) PSD length analysis reveals an increase of PSD in WT+ NGB‐2904+ 3TBS and in D3‐KO + 3TBS compared to WT + 3TBS (*p* < 0.0001 and *p* = 0.0006, respectively; Kruskal–Wallis, Dunn's test for multiple comparison) as a difference between WT + NGB‐2904 + 3TBS and D3‐KO + 3TBS (*p* = 0.0048; see Table S1 for the complete detailed analysis). Data are expressed as mean ± SEM. Scale bar: 200 nm. ****p < 0.0001, ***p < 0.001, **p < 0.01, *p < 0.05.

Based on the prevalent post‐synaptic effect of D3Rs in healthy adult mice, we investigated whether the ability of D3R blockade to restore synaptic function in aged mice relied upon changes in post‐synaptic protein expression. We performed WB experiments on hippocampal slices that previously underwent electrophysiology and found that the pharmacological blockade of D3Rs by NGB‐2904 or the genetic deletion as in D3‐KO animals restored the age‐related impairment of PSD‐95 (+117% and + 86%, respectively; Figure 6a,b), pGlua1 (+63% and + 61%, respectively; Figure 6c,d), and p‐CREB (+66% and + 97%, respectively; Figure 6e,f).

These results were corroborated by EM studies evaluating ultrastructural synaptic modifications in tetanized hippocampal slices where D3Rs were antagonized or deleted. By investigating specific ultrastructural changes (i.e., vesicle pool, number of docked vesicles, area of spines, PSD length, and percentage of perforated synapses; Table S1) (Gulisano et al., 2019) at axo‐spinous synapses of CA1 *stratum radiatum*, we observed an increase of PSD length in tetanized slices treated with the D3R antagonist NGB‐2904 and in those from D3‐KO mice (Table S1; Figure 6g,h), indicating that synapses display plasticity‐induced changes at the post‐synaptic site following D3R blockade or deletion.

## 4 DISCUSSION

**FIGURE 7:**
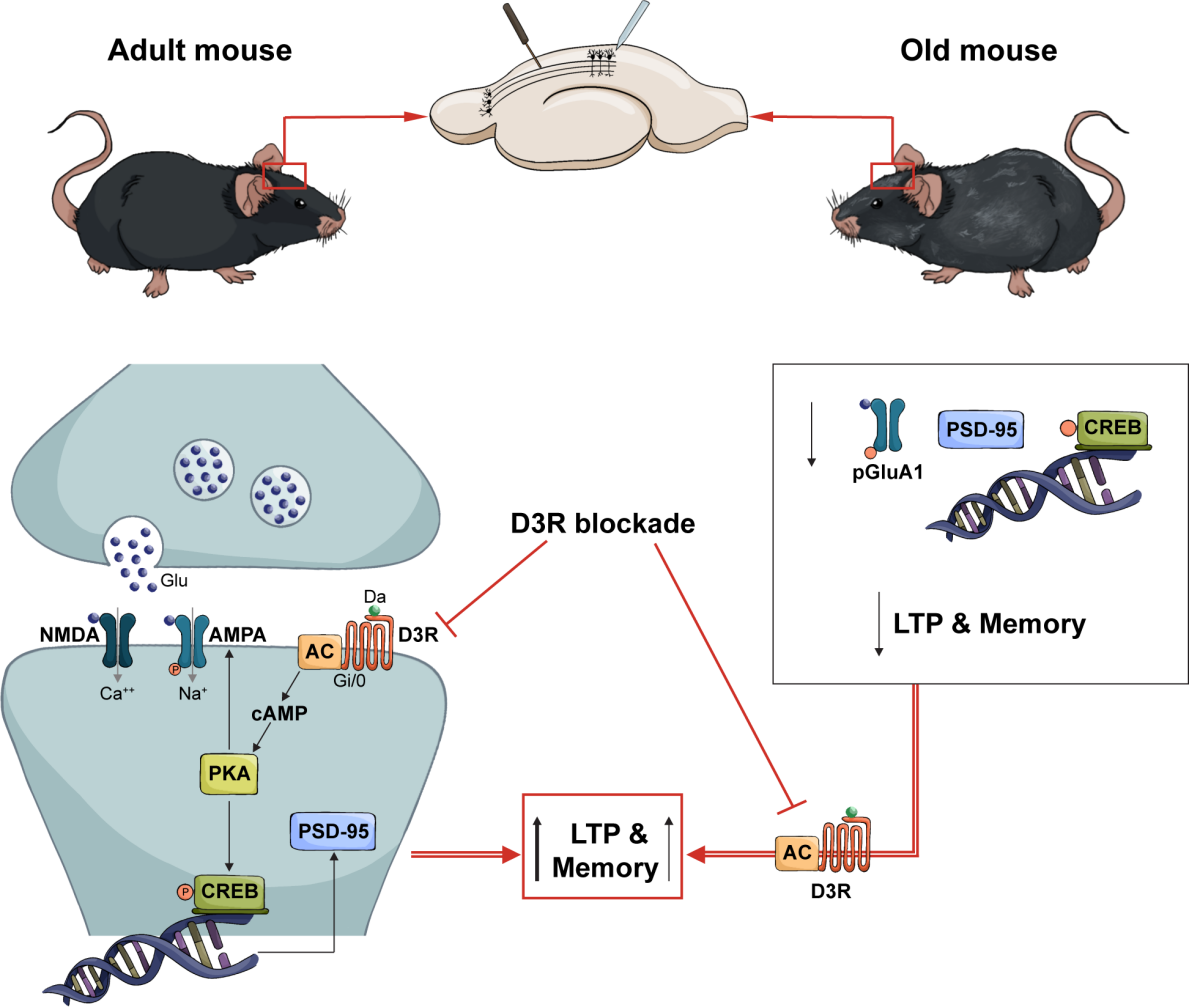
Effects of D3R blockade on synaptic plasticity and memory in adult and aged mice. Picture depicting the main results of our manuscript. In adult mice, D3R blockade operated through the cAMP/PKA pathway, leading to increased expression of phopsho(p)‐CREB, p‐GluA1 (AMPA receptor subunit), and PSD‐95. This results in structural modifications that promote long‐lasting LTP and memory formation. In old mice, D3R blockade normalized the decreased expression of post‐synaptic proteins and reversed the impairment of LTP and memory. NMDA = N‐methyl‐D‐aspartate receptors; AMPA, α‐amino‐3‐hydroxy‐5‐methyl‐4‐isoxazolepropionic acid receptors: Glu, glutamate; Da, dopamine; AC, adenylyl cyclase.

In this work we showed that pharmacological blockade or genetic deletion of D3Rs enhanced hippocampal synaptic transmission and plasticity, as well as the expression of post‐synaptic plasticity‐related proteins, and memory in adult mice. Notably, our findings also evidenced that these effects were predominantly mediated by post‐synaptic mechanisms, in line with the higher presence of D3Rs on post‐synaptic dendrites assessed through an EM study of the CA1 area of the hippocampus. Building upon these insights, we investigated whether targeting D3Rs could offer potential avenues for addressing age‐related cognitive decline. We have indeed demonstrated that D3R blockade restored the normal cognitive phenotype, and that aged D3‐KO animals showed no signs of plasticity and memory deficits. A summary of the effect of D3R blockade on synaptic plasticity and memory in adult and old mice is illustrated in Figure 7.

Our first aim was to study the impact of D3R blockade on synaptic plasticity and transmission in hippocampal glutamatergic synapses, essential for memory formation. We found that NGB‐2904, a D3R antagonist, converted LTP1 into LTP2 and further enhanced LTP2. Notably, this conversion was also evident in D3‐KO mice, which naturally exhibited LTP2 following a weak tetanic stimulation. Few other studies have investigated the effect of D3R modulation on hippocampal transmission and plasticity. For example, it has been reported that D3R agonists increased LTP via GABAergic depression (Swant et al., 2008; Swant & Wagner, 2006), with both inhibition and stimulation of PKA preventing these effects (Swant et al., 2008). However, although we cannot exclude that D3R agonists might exhibit a positive effect on plasticity depending on concentrations, doses, and duration of treatment employed, previous literature suggests that D3R stimulation impairs, while D3R blockade improves memory (reviewed in Kiss et al., 2021). This is in line with our data clearly indicating that blocking D3Rs enhanced synaptic transmission and plasticity via the cAMP/PKA pathway. Indeed, perfusion with either an adenylyl cyclase or a PKA antagonist prevented NGB‐2904 from converting LTP1 into LTP2. The results align with the recognized function of D3Rs that decrease cAMP and subsequently lessen PKA activity (Robinson & Caron, 1997). Consequently, blocking D3Rs would stimulate the cAMP/PKA pathway leading to an increase of hippocampal LTP, as extensively demonstrated.

Building on electrophysiological findings, we also thoroughly investigated the localization and distribution of D3Rs in the hippocampus. Light microscopy demonstrated a widespread presence of D3Rs across all hippocampal strata, aligning with previous studies that explored D3R localization in the hippocampus of rodents (Li & Kuzhikandathil, 2012), and humans (Meador‐Woodruff et al., 1994). Here, EM ultrastructural studies provided a detailed sub‐localization of D3Rs within the hippocampal CA1 region, revealing a high expression of D3Rs in excitatory asymmetric synapses in the pyr‐sr layers, with a differential distribution of D3 IR among post‐synaptic dendrites, axon terminals, and astrocytic processes. Interestingly, D3 IR was more prevalent in post‐synaptic dendrites than in axon terminals. This aligns with previous studies placing D3Rs in the post‐synaptic dendrites of excitatory synapses in the nucleus accumbens (Sokoloff et al., 2013), where they modulate glutamatergic transmission (Liu et al., 2009).

Our functional data converged with EM findings, suggesting that D3 antagonism primarily impacts post‐synaptic mechanisms as supported by the increase of fEPSP and mEPSC amplitude, and AMPA currents with no changes in pre‐synaptic indices (i.e., PTP, PPF, afferent volley, and mEPSC frequency). Consistently, we found an increased expression of post‐synaptic proteins such as PSD‐95, pGluA1, and pCREB in slices treated with the D3R antagonist paired with a weak tetanic stimulation as well as in slices from D3‐KO animals, even in basal conditions. These findings fit with the well‐known role of post‐synaptic proteins in synaptic and memory functions as phosphorylation of GluA1 at Ser845 boosts AMPA‐R conductance and trafficking, mediating LTP by a PKA‐dependent mechanism (reviewed in Kessels & Malinow, 2009). PSD‐95, a post‐synaptic density constituent, regulates AMPA‐R incorporation and positioning during hippocampal plasticity and memory, impacting AMPA‐R‐mediated synaptic currents (Ehrlich & Malinow, 2004). Interestingly, it has been previously demonstrated that D3Rs interact with Ca^2+^/calmodulin‐dependent protein kinase II in the PSD of glutamatergic neurons leading to a stimulation of AMPA‐R GluA1 subunit (Liu et al., 2009), initiating intracellular signaling crucial for long‐lasting LTP and memory, including CREB phosphorylation. Consistently, we found that pharmacological blockade or genetic D3R deletion enhanced pCREB aligning with previous studies demonstrating increased pCREB in D3‐KO mice neuronal cultures treated with nicotine (Mutti et al., 2022), and in hippocampal tissues of D3‐KO mice trained with passive avoidance test (D'Amico et al., 2013). In this context, the basal increase in plasticity‐related proteins mediated by D3R blockade might represent a crucial event for synaptic tagging. In fact, after a weak tetanic stimulation, synapses should enter a receptive state that, when aligns with the production of plasticity‐related proteins, supports the maintenance of LTP (Wang et al., 2010).

Taken together, our data showed that the blockade of D3Rs induced molecular and functional modifications at the synapse that are crucial for LTM formation. Consequently, we investigated their role in memory processes. We found that acute treatment with the D3Rs antagonist NGB‐2904 was able to induce LTM, exerting a robust pro‐cognitive effect on recognition and spatial memory. Interestingly, similar results were obtained with D3‐KO animals, that were capable to naturally form LTM when exposed to a short training phase. These findings are in line with previous works demonstrating that the blockade or knockout of D3Rs exerts pro‐cognitive effects (reviewed in Kiss et al., 2021). However, considering that the precise contribution of these receptors to hippocampal memory processes has not been thoroughly investigated, we injected the D3R antagonist NGB‐2904 selectively into the hippocampus and found the same effects evidenced with a systemic treatment, proving that the blockade of hippocampal D3Rs enhanced memory. Notably, the blockade of D3Rs did not modify the total exploration time in NOR and NOL tests, nor the time spent in the center during the open field test. This suggests that D3R blockade did not affect movement or thigmotaxis, the latter being an indicator of unconditioned anxiety‐like behavior. This is particularly significant considering that D3Rs are implicated in several brain functions, including emotional behavior and locomotion (reviewed in Sokoloff & Le Foll, 2017). Further studies are needed to better understand these aspects and their influence on memory.

Based on data obtained in the healthy mouse brain, we decided to investigate the effects of D3R blockade during aging and to exploit it as a possible strategy to counteract age‐related cognitive decline. First, we evaluated if aged hippocampi presented a modification of D3R quantity and distribution at hippocampal level. We demonstrated a significant decline of D3+ profiles within the hippocampal CA1 area, in agreement with earlier findings highlighting a significant age‐related decline of D3R mRNAs in CA1 pyramidal neurons in human post‐mortem brain tissues (Hemby et al., 2003). Interestingly, D3R expression remained unaltered in the post‐synaptic dendrites but decreased at pre‐synaptic sites. This imbalance might lead to reduced regulation or control over the quantity or timing of dopamine release and could heighten sensitivity or responsiveness of the receiving neuron to dopamine signaling thus altering downstream signaling pathways that influence synaptic transmission and plasticity.

In this context, the positive effects of D3R blockade on age‐related cognitive decline could stem from restoring this imbalance. Specifically, treatment with two distinct D3R antagonists rescued impairment in synaptic transmission and plasticity in slices from aged mice. Conversely, D3‐KO animals appeared resistant to aging, displaying no impairment in synaptic plasticity and transmission. NGB‐2904 also completely restored the impairment of recognition memory and spatial learning and memory in aged mice, whereas D3‐KO mice did not show any of the age‐related memory impairment. These results are consistent with a previous work demonstrating that aged D3‐KO mice maintained intact spatial memory compared to aged‐matched WT (Xing et al., 2010). Furthermore, they align with other studies showing the pro‐cognitive effect of D3Rs in models of cognitive impairment (Laszy et al., 2005; Millan et al., 2007; Neill et al., 2016; Torrisi et al., 2017; Zimnisky et al., 2013).

Considering that previous studies reported sex differences in D3‐KO behavior (Klinker et al., 2017; Liu et al., 2017), and given the disproportionate impact of cognitive decline in the hippocampus on the aging female population, we analyzed our behavioral data for potential sex differences. However, neither adult nor old animals exhibited any sex differences, whether they were WT, treated with NGB‐2904, or D3‐KO mice. It is important to note that our analysis was conducted on existing sex‐balanced data divided by sex. Given the relatively low numbers of males and females, this could potentially mask some differences and might deserve further investigations.

In line with prior research, aged mice exhibited a decline in post‐synaptic proteins associated with synaptic strengthening and memory, including pGluA1‐Ser845 (Henly and Kilkinson, 2013), PSD‐95 (Zarate et al., 2021), and pCREB‐Ser133 (Palmeri et al., 2013). Corroborating our electrophysiological and behavioral findings, we observed that treatment with NGB‐2904 restored their expression in hippocampal slices from aged mice following tetanization. Intriguingly, slices from D3‐KO animals exhibited elevated levels compared to age‐matched WT mice, even without tetanic stimulation. Furthermore, EM performed on hippocampal slices from aged WT mice showed that pharmacological blockade of D3Rs induced structural modifications at the post‐synaptic site consisting in an increase of PSD lenght, a known factor underlying functional long‐lasting synaptic potentiation.

In conclusion, our data contribute to clarifying the role of D3Rs at hippocampal glutamatergic synapses and suggest the potential use of D3R antagonists as pharmacological agents for treating or preventing memory loss associated with physiological aging and neuropsychiatric disorders characterized by memory decline, holding high translational applicability.

## AUTHOR CONTRIBUTIONS

M.R.T., M.M., D.D.L.P., V.V., G.A., B.B., and R.C.T. conducted the experiments; M.R.T., M.M., D.D.L.P., G.A., and D.P. analyzed the data; M.R.T., M.M., S.A.T., G.M.L., A.P., M.D.A., F.C., C.G., and D.P. interpreted results. M.R.T. and D.P. conceptualized the study; all the authors contributed to write the manuscript. D.P. supervised the study.

## FUNDING INFORMATION

This work was supported by Italian Ministry of University and Research PRIN 2020AMLXHH to M.M., C.G. and D.P., PRIN 2022BZWEK to F.C., PRIN 2022YEPFB7 to M.D.A. and D.P.; Italian Ministry of HealthRicerca Corrente (Fondazione Policlinico Universitario A. Gemelli IRCCS to C.G., Oasi Research Institute IRCCS to D.P.), University of Catania intramural funds “Progetto Piaceri” to D.P.

## CONFLICT OF INTEREST STATEMENT

The authors declare no competing interests.

## Supporting information


**Data S1:** Supporting Information.

## Data Availability

The data that support the findings of this study are available from the corresponding author upon reasonable request.
