# Supplementary material for: Blockade of dopamine D3 receptors improves hippocampal synaptic function and rescues age‐related cognitive phenotype
Source: Aging Cell. 2024 Sep 5;23(11):e14291. doi: 10.1111/acel.14291 (PMC11561665; doi:10.1111/acel.14291)
Supplement: Supplementary file 1 — Data S1: Supporting Information. [file ACEL-23-e14291-s001.docx]

**SUPPLEMENTARY INFORMATION**

**Blockade of dopamine D3 receptors improves hippocampal synaptic function and rescues age-related cognitive phenotype**

Maria Rosaria Tropea, Marcello Melone, Domenica Donatella Li Puma, Valeria Vacanti, Giuseppe Aceto, Bruno Bandiera, Roberta Carmela Trovato, Sebastiano Alfio Torrisi, Gian Marco Leggio, Agostino Palmeri, Marcello D’Ascenzo, Fiorenzo Conti, Claudio Grassi, Daniela Puzzo

**SUPPLEMENTARY METHODS**

**Electrophysiology**

Electrophysiological field recordings

Extracellular field recordings were conducted on 400 μm transverse hippocampal slices as described previously (Gulisano et al., 2019). After sectioning using a manual tissue chopper, the slices were placed in a recording chamber and perfused (1–2 ml/min) with ACSF solution containing (in mM) 124 NaCl, 4.4 KCl, 1 Na2HPO4, 25 NaHCO3, 2 CaCl2, 2 MgCl2, and 10 glucose, maintained at 29°C and continuously bubbled with 95% O2 and 5% CO2. After 120 minutes of recovery, field excitatory post-synaptic potentials (fEPSPs) were recorded in CA1 stratum radiatum (*sr*) using a glass electrode filled with ACSF in response to Schaffer collateral stimulation by a bipolar tungsten electrode.

Basal synaptic transmission (BST) was measured by stimulating with a series of increasing voltage pulses (from 5 to 35 V) to select healthy slices for recordings. Before LTP induction, baseline responses were recorded every minute using a voltage that evoked a response of 35% of the maximum evoked response in BST. We used a theta-burst stimulation (TBS) protocol to induce LTP where each tetanus consisted in 3 trains of 10 bursts at 100 Hz, with five pulses per burst repeated at 5 Hz. We have studied LTP1 and LTP 2, frequently referred to as early-phase LTP and late-phase LTP, corresponding to forms of LTP that are, respectively, independent of and dependent on de novo protein synthesis (Bliss et al., 2018). To induce LTP1 a single TBS train (weak tetanic stimulation) was delivered, while for LTP2, three TBS trains were delivered with a 15-second intertrain interval (strong tetanic stimulation). fEPSP slopes were normalized to the first 15 minutes of baseline recordings.

In additional experiments we evaluated the presynaptic component of post-tetanic potentiation (PTP) and paired-pulse facilitation (PPF) in the presence of the NMDA receptor antagonist (2R)-amino-5-phosphonovaleric acid (APV; 50 μM) applied for 45 minutes before recordings. For PTP, we delivered three 10-burst trains similar to those used to produce LTP. For PPF, paired pulses were delivered with varying time intervals (10, 20, 30, 40, 50, 100, 200, 500, and 1000 ms), and the percentage of the synaptic response of the second stimulus against the first delivered stimulus was recorded. All the recordings were performed and analyzed using pClamp10.

Patch-clamp recordings

Brain slices were prepared as previously described (Aceto et al., 2022), with minor modifications. Slices (350 μm thick) were cut on a vibratome (VT1200S; Leica Microsystems, Germany) and immediately transferred to an incubation chamber held at 32°C and filled with a recovery solution. After 30 min, slices were transferred to a second incubation chamber held at 32°C and filled with ACSF containing (in mM): 124 NaCl, 3.2 KCl, 1.2 NaH_2_PO_4_, 1 MgCl_2_, 2 CaCl_2_ 2, 26 NaHCO_3_ and 10 glucose, pH 7.4. Slices were equilibrated at RT for at least 45 min and then transferred to a submerged recording chamber constantly perfused with heated ACSF (32°C). Slices were constantly bubbled with 95% O_2_/5% CO_2_. Neurons of the CA1 area were visualized under DIC infrared illumination. Stimulation of the SC was obtained by means of a current stimulus isolator (WPI, Worcester, MA, USA), connected to a bipolar concentric stimulating electrode (FHC, Bowdoin, ME, USA) which was positioned in contact with the SC pathway. Patch pipettes had a resistance of 4–6 MΩ when filled with an internal solution containing (in mM): 145 K-gluconate, 2 MgCl_2_, 10 HEPES, 0.1 EGTA, 2.5 Na-ATP, 0.25 Na-GTP, 5 phosphocreatine, pH adjusted to 7.2 with KOH. For AMPA/NMDA ratio experiments, the internal solution contained (in mM): 135 CsCH_3_SO_3_, 10 HEPES, 8 NaCl, 0.25 EGTA, 2 MgCl_2_, 4 Mg-ATP, 0.3 Na-GTP, 5 phosphocreatine, pH adjusted to 7.3 with NaOH. After establishing a gigaseal, the patch was broken by applying negative pressure to achieve a whole-cell configuration. A series resistance lower than 15 MΩ was considered acceptable and monitored constantly throughout the entire recording. For evoked and spontaneous Excitatory Post-synaptic Currents (EPSC) measurements, neurons were held at −70 mV and electrical stimuli were delivered to the SC. To obtain the AMPA/NMDA currents ratio, stimuli of identical amplitude were delivered at holding potentials of −70 and +40 mV, as described (Ahmad et al., 2012), with a frequency of 0.05 Hz; 50 μM picrotoxin (PTX) was added to the bath. The identity of evoked EPSCs was confirmed at the end of the recordings by adding 10 μM of the selective AMPA receptor blocker 2,3-Dioxo-6-nitro-1,2,3,4-tetrahydrobenzo[*f*]quinoxaline-7-sulfonamide (NBQX) to the bath. For miniature EPSCs (mEPSCs) measurements, 0.5 μM tetrodotoxin and 50 μM PTX were applied to the bath. Recordings were performed in cells from slices incubated (10-20 min) with either vehicle or NGB-2904 (1 μM). Recordings were performed using a Multiclamp 700B/Digidata 1550A system (Molecular Devices, Sunnyvale, CA, USA) and digitized at a 10,000 Hz sampling frequency. All the electrophysiological recordings were analyzed using the Clampfit 10.6 software (Molecular Devices). AMPA receptor-mediated EPSC amplitude was calculated as the difference between the peak response and the baseline. NMDA-receptor-mediated EPSC amplitude was calculated as the amplitude 50 ms (Ahmad et al., 2012) after the response onset. For mEPSC frequency analysis, a template was constructed using the “Event detection/create template” function, as described (Leggio et al., 2019). Then, mEPSCs were detected using the “Event detection/template search” function and the result inspected for false positives. For mEPSC amplitude analysis, all the waveforms detected during a single recording using template analysis were averaged and the amplitude calculated.

**Electron microscopy**

Animals and tissue preparation

Three 8-month-old, three 21-month-old male C57BL/6 mice, and four 7-8-month-old mice (two WT and two D3-KO) mice were anesthetized with an intraperitoneal injection of chloral hydrate (300 mg/kg) and perfused transcardially with a flush of saline solution, followed by 4% freshly depolymerized paraformaldehyde and 0.2% glutaraldehyde in 0.1 M phosphate buffer (PB; pH 7.4). Brains were removed, post-fixed in the same fixative (for 48 hrs) and cut on a Vibratome in 50 µm serial parasagittal sections, which were collected in PB until processing.

Immunoperoxidase and pre-embedding procedures

Sections were pre-treated with sodium borohydride for 20 min to prevent non-specific binding, treated with H2O2 (1% in PB; 30 min) to remove endogenous peroxidase activity, rinsed in PB and pre-incubated in 10% normal donkey serum (NDS, 1 hr). According to (Requie et al., 2022), sections were then incubated in a solution containing anti-D3 primary antibodies (1:100; ADR-003, RRID:AB_2039830; Alomone, rabbit primary antibodies raised against a synthetic peptide corresponding to AA 15-29 within the extracellular N-terminus sequence of rat D3 receptor validated by the lack of immunoreactivity in D3 knock-out mice; Castro et al., 2015; Figure S1); 2 hr at room temperature [RT] and overnight at 4°C. The following day, sections were rinsed 3 times in PB and incubated first in 10% NDS (15 min) and then in a solution containing secondary biotinylated secondary antibodies (1:300; Jackson; 1.5 hr at RT). Sections were subsequently rinsed in PB, incubated in avidin-biotin peroxidase complex (ABC Elite PK6100, Vector), washed several times in PB, and incubated in 3,3’diaminobenzidine tetrahydrochloride (DAB; 0.05% in 0.05 M Tris buffer, pH 7.6 with 0.03% H_2_O_2_). Method specificity was verified by substituting primary antibodies with PB. As previously described (Melone et al., 2019) after completion of immunoperoxidase procedures, sections were post-fixed in 1% osmium tetroxide in PB for 45 m and contrasted with 1% uranyl acetate in maleate buffer (pH 6.0; 1 h). After dehydration in ethanol and propylene oxide, sections were embedded in Epon/Spurr resin (Electron Microscopy Sciences, Hatfield, PA, USA), flattened between Aclar sheets (Electron Microscopy Sciences) and polymerized at 60°C for (48 hrs). Chips including *sr* of CA1 were selected by light-microscopic inspection, glued to blank epoxy and sectioned with an ultramicrotome (MTX; Research and Manufactoring Company Inc., Tucson, AZ, USA). The most superficial ultrathin sections (~60 nm) were collected and mounted on 300 mesh nickel grids, stained with Sato’s lead and examined with a Philips EM 208 and CM100 electron microscopes coupled to a MegaView-II high resolution CCD camera (Soft Imaging System). To minimize the effects of procedural variables, all material from 8 and 21 mice was processed in parallel.

Electron microscopy of hippocampal slices

Hippocampal slices (n = 2 for each condition from 8 old animals) were immersed (within ~ 30 s after 120 min of electrophysiological recording) in a solution containing 4% paraformaldehyde and 0.5% glutaraldehyde in phosphate buffer (PB) and then stored for 12 weeks at 4°C in the same fixative solution. Next, slices were embedded procedure according to previous studies (Gulisano et al., 2019; Melone et al., 2019) and the protocol applied for pre-embedding procedures described above. A small block of slices containing CA1 *sr* was selected and sectioned with an ultramicrotome; 18-20 ultrathin sections (~60 nm) for each CA1 block were mounted on 200 mesh copper grids, contrasted, and examined.

Data collection and electron microscopical analysis of D3 pre-embedded material

All data were obtained from *sr* CA1 of immunoreacted sections. D3 immunoreactive profiles were studied in ultrathin sections from the surface of the embedded blocks. Quantitative data derived from the analysis of microscopic fields (10–12 ultrathin sections/animal) selected and captured at original magnifications of 25,000x-30,000x. Microscopical fields containing positive processes were randomly selected. Acquisition of microscopical fields of 8- and 21-months-old mice was performed under blinded conditions (167 fields/3 mice from 8-month-old animals and 173 fields/3 mice from 21-month-old mice). For the analysis of the density of D3 positive profiles (characterized by the presence of immunoreactive electron dense products), subcellular compartments were identified according to well-established criteria (Peters et al., 1991). For quantifying D3 distribution at asymmetric synapses, the identification of synaptic domains was based according to previous studies (DeFelipe et al., 1999; Requie et al., 2022; Melone et al., 2019). Pre-synaptic axon terminals were characterized by the presence of clear and round vesicles nearby the pre-synaptic density (active zone) associated with a synaptic cleft displaying electrodense material, post-synaptic elements showed a prominent post-synaptic density typical of the asymmetric synapses (PSD), and astrocytic profiles in close relationship with neuronal synaptic elements, were identified based on their typical irregular outlines and the paucity of cytoplasmic components (with the exception of ribosomes, glycogen granules and various fibrils).

Data collection and electron microscopical analysis of slices

Electron microscopy was performed on hippocampal slices from aged WT treated with three TBS, with NGB and three TBS, and on hippocampal slices from aged D3KO mice treated with three TBS. Quantitative analysis of vesicle pool, number of docked vesicles, area of spines, length of the post-synaptic density (PSD), and the proportion of perforated synapses was carried out as described (Babits et al., 2016; Bourne et al., 2013; Gulisano et al., 2019). Randomly selected electron microscopical fields of the *sr* (WT+3TBS n = 99, WT+NGB+3TBS n = 100, D3KO+3TBS n = 99) with at least an identifiable axo-spinous synapse were acquired at original magnification of 44,000x-46,000x.

*Sr* axo-spinous synapses were identified according to the criteria used for D3-prembedding studies described above. The distinction between vesicle pools (which comprise the reserve vesicle pool) and docked vesicles (which are thought to be part of the readily releasable pool), was performed as described (Bourne et al., 2013; Gulisano et al., 2019). Briefly, vesicle pools were determined by counting the total number of small vesicles (∼50 nm) per terminal; the docked vesicles pool by identifying and counting the vesicles touching the membrane of the pre-synaptic active zone. Spines profile area and PSD length were measured by Image J software tools measurement (Babits et al., 2016; Gulisano et al., 2019). Spine profiles and PSD were traced along the membranes and measured; PSD length corresponded to the distance between the edges of PSD. Perforated synapses were identified based on the presence of a discontinuous PSD (Babits et al., 2016). Ultrathin sections and microscopical features of axo-spinous synapses were examined and analyzed blinded.

**Behavioral studies**

Hippocampal cannulas implantation

Mice underwent stereotaxic surgery for intrahippocampal cannulas implantation. After anesthesia with tiletamine + zolazepam (60 mg/kg) and medetomidine (40 μg/kg), mice were implanted with a 26-gauge guide cannula into the dorsal part of the hippocampi (coordinates from bregma: posterior = 2.46 mm, lateral = 1.50 mm to a depth of 1.30 mm). The cannulas were fixed to the skull with acrylic dental cement (RelyX^TM^ Unicem, 3M), and mice were allowed to recover for a minimum of 6-8 days. Fifteen minutes before the training phase (T1) of the novel object recognition test, mice were bilaterally infused with NGB-2904 (1 μM) in a final volume of 1 μl over 1 min, using a microsyringe connected to the cannulas via polyethylene tubing. During infusion, animals were handled gently to minimize stress. After infusion, the needle was left in place for another minute to allow diffusion. In some animals, after behavioral studies, a solution of 4% methylene blue was infused for localization of infusion cannulas (Tropea et al., 2022a).

Open field

Open Field was performed in an apparatus of our design and construction, consisting of an arena (45 × 45 × 40 cm) made by white matte polymethyl methacrylate non-reflective panels illuminated by a perpendicular light source located 65 cm from the floor (Tropea et al., 2022b). A webcam, connected to the computer, was fixed on the top of the apparatus. Each mouse was placed in the arena and allowed to freely explore for 5 min. We scored the percentage of time spent in the center and the number of entries into the center.

Novel Object Recognition and Location

Novel Object Recognition (NOR) and Novel Object Location (NOL) tests were performed as previously described (Gulisano et al., 2019; Tropea et al., 2021) using the same apparatus utilized for open field experiments. After 5 days of habituation to the arena, objects, and i.p. injections, mice underwent the training session (T1), and after 24 hrs, the testing session (T2) was conducted to assess memory retention. We used two different protocols: i) to assess whether D3R blockade enhanced cognition in young mice we employed a “short protocol” (T1 = 3 minutes, T2 = 5 minutes), which is known to induce short-term memory that typically fades after about 6-8 hours (Blokland and Sesia, 2023). Consequently, when testing memory after 24 hours, the animals normally cannot distinguish between the familiar objects presented during T1 and the new object in T2; ii) to evaluate long-term memory, we used a “long protocol” (T1 = 10 minutes, T2 = 10 minutes) since the longer exposure during training allows for memory retention that can be assessed after 24 hours. The “long protocol” has been utilized to determine whether D3R blockade could restore memory in old animals or further enhance memory formation in young mice. In both protocols, during T1, mice were placed in the arena and allowed to explore two identical objects. In T2, for NOR, mice were presented with two different objects, a "familiar" one (i.e., the one used in T1) and a "novel" one; for NOL, mice were presented with an object located as in T1, and the other in a different location. For both tests, animal exploration - defined as the mouse pointing its nose toward the object from a distance not > 2 cm - was measured in T2. We analyzed discrimination (D) index, “exploration of novel object minus exploration of familiar object/total exploration time”, and total exploration time. Mice with a total exploration time < 5 s were excluded from analysis.

Morris Water Maze

Morris Water Maze was performed as previously described (Gulisano et al., 2018) in a plastic maze filled with water (25°C) made opaque by the addition of nontoxic white paint. The submerged platform was located in the south-west quadrant and spatial cues were placed on the 4 cardinal points of the maze. Spatial learning was assessed over 5 days (2 daily sessions, 3 trials of 1 min each). Mice started from a randomly chosen quadrant, and the time taken to reach the hidden submerged platform (latency) was recorded. The 6^th^ day, to evaluate reference memory retention, the probe test was performed (one session, 4 trials of 1 min each). The maze was divided into 4 quadrants (target quadrant [TQ] previously containing the platform, adjacent left, adjacent right, and opposite quadrant) and the percent of time spent in each quadrant was recorded and analyzed with a video tracking system (Netsense srl, Catania, Italy). Visual, motor, and motivation skills were assessed by measuring the time taken to reach a visible platform randomly positioned in different places and marked with a green flag (2 daily sessions, 3 trials of 1 min each). For the analyses, results from trials performed in each session were averaged.

**REFERENCES**

1. Aceto, G., Nardella, L., Nanni, S., Pecci, V., Bertozzi, A., Colussi, C., D'Ascenzo, M., & Grassi C. (2022). Activation of histamine type 2 receptors enhances intrinsic excitability of medium spiny neurons in the nucleus accumbens. *Journal of Physiology*. **600**, 2225-2243. doi: 10.1113/JP282962.
2. Ahmad, M., Polepalli, J.S., Goswami, D., Yang, X., Kaeser-Woo, Y.J., Südhof, T.C., & Malenka, R.C. (2012). Postsynaptic complexin controls AMPA receptor exocytosis during LTP. *Neuron*. **73**, 260-267. doi: 10.1016/j.neuron.2011.11.020.
3. Babits, R., Szőke, B., Sótonyi, P. & Rácz, B. (2016). Food restriction modifies ultrastructure of hippocampal synapses. *Hippocampus*. **26**, 437-44. doi: 10.1002/hipo.22533.
4. Blokland, A., & Sesia, T. (2023). Delay-dependent forgetting in object recognition and object location test is dependent on strain and test. Behavioural Brain Research. 437, 114161. doi: 10.1016/j.bbr.2022.114161.
5. Bourne, J.N., Chirillo, M.A., & Harris, K.M. (2013). Presynaptic ultrastructural plasticity along CA3→CA1 axons during long-term potentiation in mature hippocampus. *Journal of Comparative Neurology*. **521**, 3898-3912. doi: 10.1002/cne.23384.
6. Castro-Hernández, J., Afonso-Oramas, D., Cruz-Muros, I., Salas-Hernández, J., Barroso-Chinea, P., Moratalla, R., Millan, M.J. & González-Hernández, T (2015). Prolonged treatment with pramipexole promotes physical interaction of striatal dopamine D3 autoreceptors with dopamine transporters to reduce dopamine uptake. *Neurobiology of Disease*. **74**, 325-35. doi: 10.1016/j.nbd.2014.12.007.
7. DeFelipe, J., Marco, P., Busturia, I., & Merchán-Pérez, A. (1999). Estimation of the number of synapses in the cerebral cortex: methodological considerations. *Cerebral Cortex*. **9**, 722-732. doi: 10.1093/cercor/9.7.722.
8. Gulisano, W., Melone M, Ripoli C, Tropea MR, Li Puma DD, Giunta S, Cocco S, Marcotulli D, Origlia N, Palmeri A, Arancio O, Conti F, Grassi C, & Puzzo D. (2019). Neuromodulatory Action of Picomolar Extracellular Aβ42 Oligomers on Presynaptic and Postsynaptic Mechanisms Underlying Synaptic Function and Memory. *Journal of Neuroscience*. **39**, 5986-6000. doi: 10.1523/JNEUROSCI.0163-19.2019.
9. Gulisano, W., Tropea, M.R., Arancio, O., Palmeri, A., & Puzzo, D. (2018). Sub-efficacious doses of phosphodiesterase 4 and 5 inhibitors improve memory in a mouse model of Alzheimer's disease. *Neuropharmacology*. **138**, 151-159. doi: 10.1016/j.neuropharm.2018.06.002.
10. Leggio, G.M., Di Marco, R., Gulisano, W., D'Ascenzo, M., Torrisi, S.A., Geraci, F., Lavanco, G., Dahl, K., Giurdanella, G., Castorina, A., Aitta-Aho, T., Aceto, G., Bucolo, C., Puzzo, D., Grassi, C., Korpi, E.R., Drago, F., & Salomone, S. (2019). Dopaminergic-GABAergic interplay and alcohol binge drinking. *Pharmacological Research*. **141**, 384-391. doi: 10.1016/j.phrs.2019.01.022.
11. Melone, M., Ciriachi, C., Pietrobon, D., & Conti, F. (2019). Heterogeneity of astrocytic and neuronal GLT-1 at cortical excitatory synapses, as revealed by its colocalization with Na+/K+-ATPase α isoforms. *Cerebral Cortex*. **29**, 3331-3350. doi: 10.1093/cercor/bhy203.
12. Peters, A., Palay, S.L., & Webster H. deF. (1991). The fine structure of the nervous system: The neurons and supporting cells (Oxford University Press, New York)
13. Requie, L.M., Gómez-Gonzalo, M., Speggiorin, M., Managò, F., Melone, M., Congiu, M., Chiavegato, A., Lia, A., Zonta, M., Losi, G., Henriques, V.J., Pugliese, A., Pacinelli, G., Marsicano, G., Papaleo, F., Muntoni, A.L., Conti, F., & Carmignoto, G. (2022). Astrocytes mediate long-lasting synaptic regulation of ventral tegmental area dopamine neurons. *Nature Neuroscience*. **25**, 1639-1650. doi: 10.1038/s41593-022-01193-4.
14. Tropea, M.R., Li Puma, D.D., Melone, M., Gulisano, W., Arancio, O., Grassi, C., Conti, F., & Puzzo, D. (2021). Genetic deletion of α7 nicotinic acetylcholine receptors induces an age-dependent Alzheimer's disease-like pathology. *Progress in Neurobiology*. **206**, 102154. doi: 10.1016/j.pneurobio.2021.102154.
15. Tropea, M.R., Sanfilippo, G., Giannino, F., Davì, V., Gulisano, W., & Puzzo, D. (2022a). Innate preferences affect results of Object Recognition Task in Wild Type and Alzheimer's disease mouse models. *Journal of Alzheimer’s Disease*. **85**,1343-1356. doi: 10.3233/JAD-215209.
16. Tropea, M.R., Torrisi, A., Vacanti, V., Pizzone, D., Puzzo, D., & Gulisano, W. (2022b). Application of 3D Printing Technology to Produce Hippocampal Customized Guide Cannulas. *eNeuro*. **9**, ENEURO.0099-22.2022. doi: 10.1523/ENEURO.0099-22.2022.

**SUPPLEMENTARY FIGURES**

**
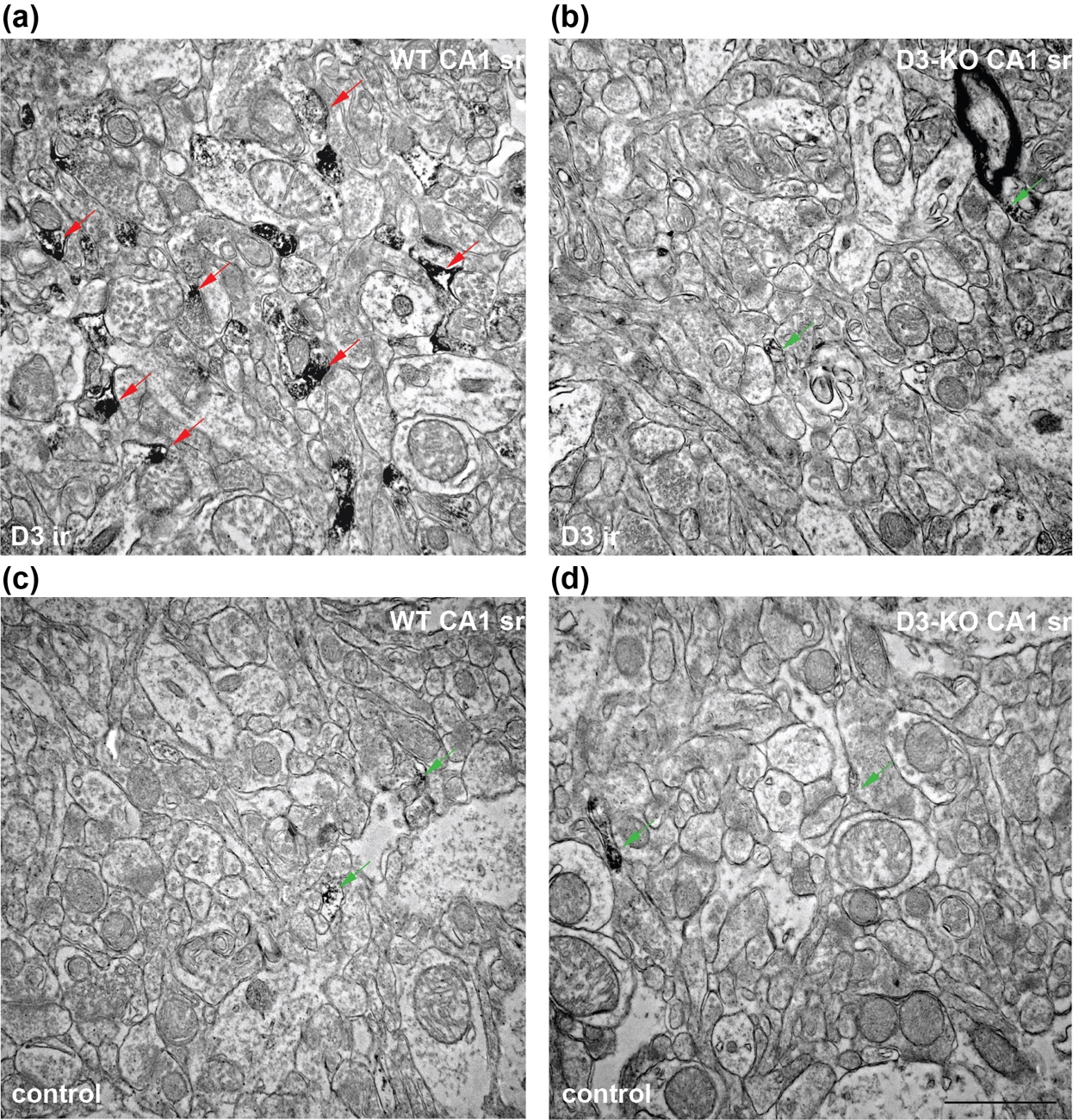
**

**FIGURE S1 – Specificity of D3 immunoreactivity in CA1 stratum radiatum. (a)** Electron microscopy of pre-embedded immunoreacted ultrathin sections revealed D3 immunoreactivity in slices from WT (red arrows) but not **(b)** D3-KO mice. Sporadic electron-dense products (green arrows) dispersed in the neuropil processes were found in D3-KO and **(c-d)** control sections reacted in parallel with the omission of D3 primary antibody. Tissue preparation, immunoperoxidase processes with the D3 primary antibody, (ADR-003, RRID:AB_2039830; Alomone), and pre-embedding methods were described in Material and Methods. Scale bar: 1 µm.

**
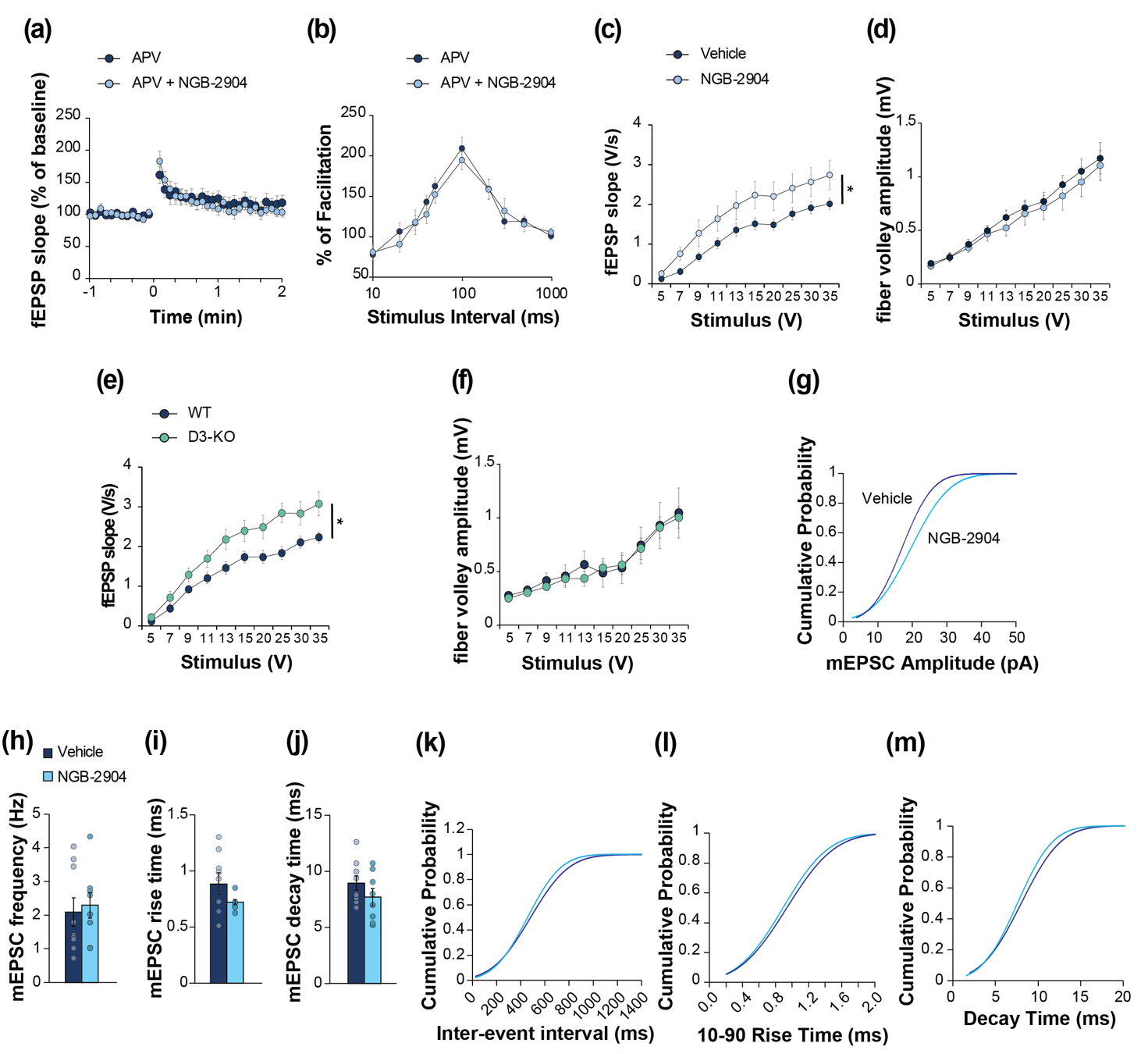
**

**FIGURE S2 – D3R blockade did not affect presynaptic forms of hippocampal synaptic transmission and plasticity.** **(a)** Perfusion of hippocampal slices with the D3R antagonist NGB-2904 (1 μM) for 15 min before tetanus did not modify post-tetanic potentiation (F_(1,20)_ = 0.11, p = 0.743, n = 10/12). **(b)** Perfusion with NGB-2904 in the presence of the NMDA receptor antagonist (2R)-amino-5-phosphonovaleric acid (APV; 50 μM) did not modify paired pulse facilitation (F_(1,19)_ = 0.434, p = 0.518, n = 11/10). **(c)** Basal synaptic transmission (BST) shown in Figure 1C plotted as fEPSP slope and **(d)** fiber volley amplitude versus stimulus intensity in NGB-2904- and vehicle-treated slices. **(e)** BST shown in Figure 1D plotted as fEPSP slope and **(f)** fiber volley amplitude in slices from D3-KO and WT animals. **(g)** Cumulative frequency distributions for mEPSC amplitude in the experimental conditions shown in Figure 1f. **(h)** Bar Graph showing that NGB-2904 did not affect mEPSC frequency (t_(15)_ = 0.354, p = 0.727), **(i)** rise time (t_(15)_ = 1.556, p = 0.140) and **(j)** decay time (t_(15)_ = 0.244, p = 0.232). **(k)** Cumulative probability graphs for mEPSC inter-invent intervals, **(l)** rise time and **(m)** decay time. Data expressed as mean ± SEM. *p< 0.05.

**
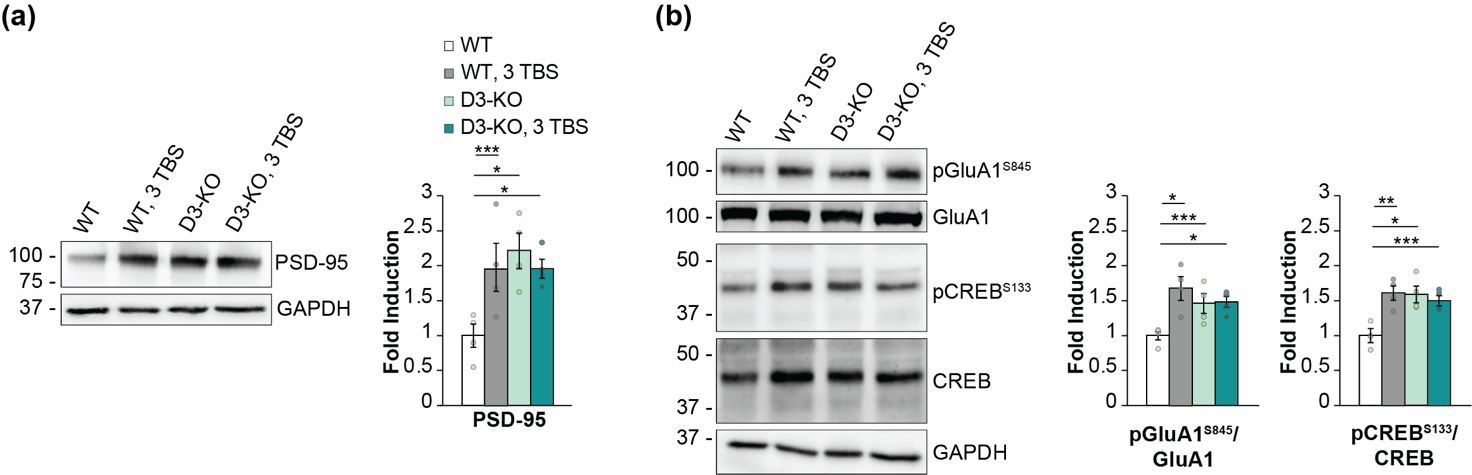
**

**FIGURE S3 – Expression of post-synaptic proteins PSD-95, pGluA1 and pCREB in slices from D3-KO mice that underwent 3 TBS.** **(a)** Representative WB images of PSD-95 (cropped images based on MW) performed in lysates from slices treated for electrophysiology stored at 1 min after 3 TBS tetanus (n = 3 slices/lane). GAPDH was used as loading control. On the right, bar graph showing the increase of PSD-95 expression in D3-KO slices in basal conditions (p = 0.046) and after 3 TBS (p = 0.012) as well as in WT slices after 3 TBS (p < 0.001). **(b)** pGluA1 and pCREB expression increased in D3-KO slices in basal conditions (p < 0.001; p = 0.046) and after 3 TBS (p = 0.012; p = 0.046) as well as in WT slices after 3 TBS (p = 0.016; p = 0.002). Data expressed as mean ± SEM. *p< 0.05; ***p< 0.01; ***p< 0.001.


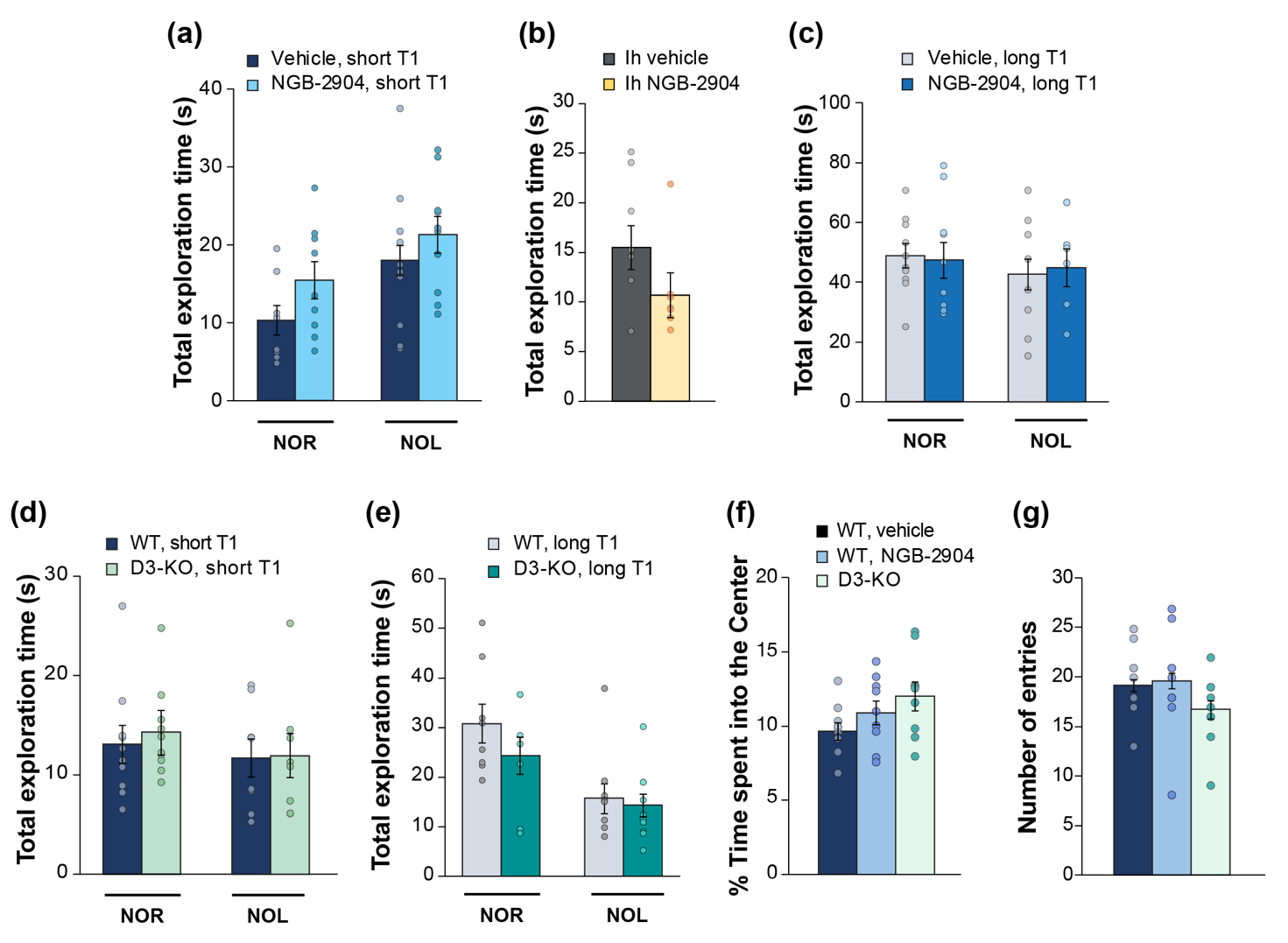


**FIGURE S4 – D3R blockade or genetic deletion did not modify exploratory behavior. (a)** Treatment with NGB-2904 (3 mg/kg, via i.p.) 20 min before a short training (T1) did not modify total exploration time in NOR (t_(15)_ = 1.659, p = 0.118) or NOL tests (t_(18)_ = 0.884, p = 0.389). **(b)** Intrahippocampal injections of NGB-2904 did not affect total exploration time (t_(13)_ = 1.745, p = 0.105). **(c)** Treatment with NGB-2904 did not modify total exploration time in mice that underwent a long T1 (NOR: t_(21)_ = 0.137, p = 0.893; NOL: t_(12)_ = 0.271, p = 0.791). **(d)** Total exploration time was unchanged in D3-KO mice after a short T1 (NOR: t_(18)_ = 0.499, p = 0.623; NOL: t_(14)_ = 0.078, p = 0.939) or **(e)** a long T1 (NOR: t_(14)_ = 1.190, p = 0.254; NOL: t_(17)_ = 0.360, p = 0.723). **(f)** Open field showed no differences in WT treated with vehicle or NGB-2904 and D3-KO mice (n = 9 for each condition) testing in the percentage of time spent in the center compartment (F_(2,24)_ = 2.224, p = 0.13) and **(g)** the number of entries into the center compartment (F_(2,24)_ = 1.114, p = 0.345). Data expressed as mean ± SEM.


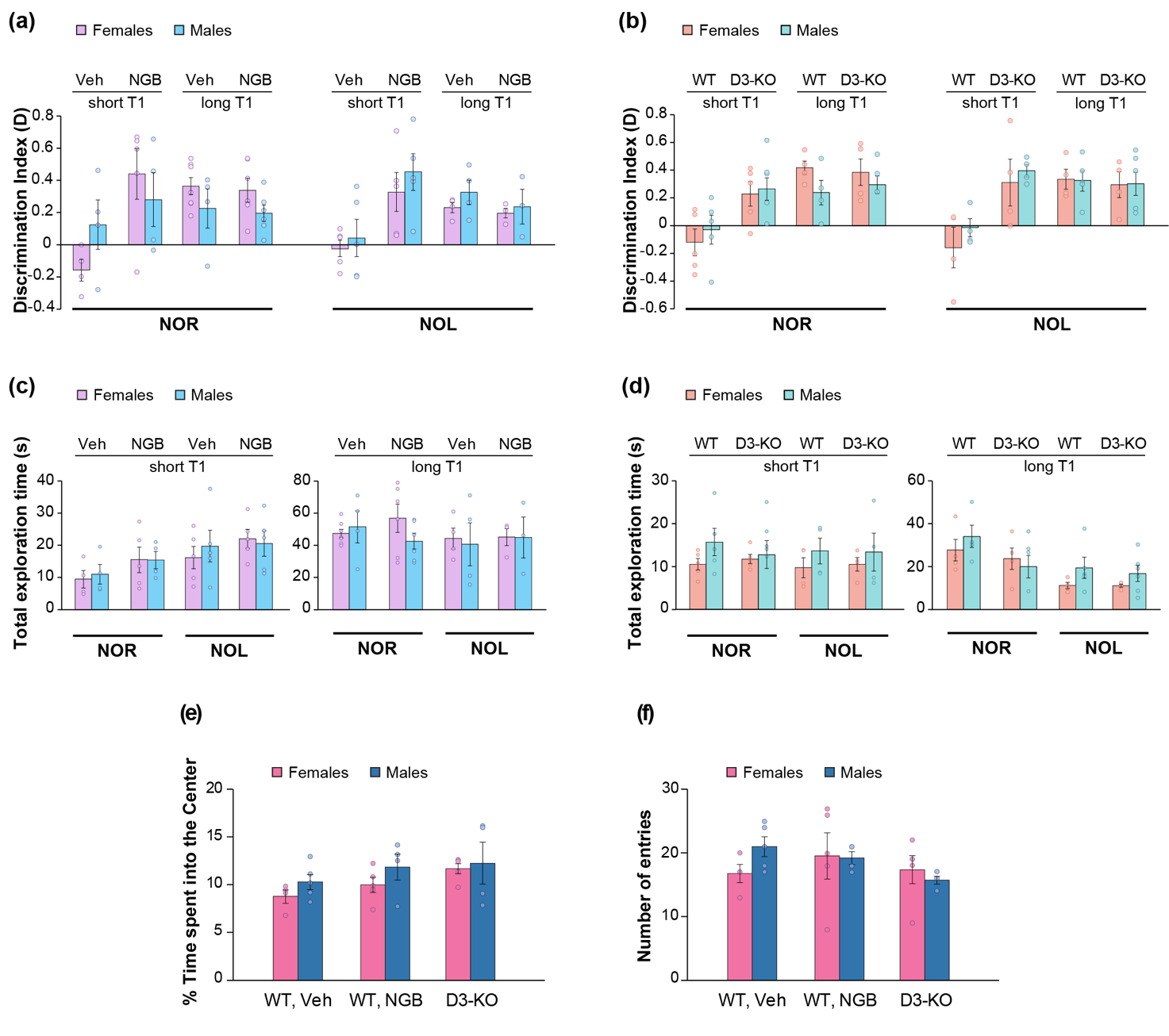


**FIGURE S5 – Sex did not influence the effects of D3R blockade in behavioral studies in adult mice. (a)** No sex differences were detected in discrimination index evaluated by NOR or NOL in vehicle- and NGB-2904-treated young mice that underwent a short or a long T1 (NOR short: vehicle t_(6)_ = 1.68, p = 0.144, n = 4F/4M; NGB-2904: t_(7)_ = 1.69, p = 0.509, n = 5F/4M; NOR long: vehicle t_(9)_ = 1.21, p = 0.256, n = 7F/4M; NGB-2904: t_(10)_ = 1.69, p = 0.120, n = 6F/6M; NOL short: vehicle t_(8)_ = 0.51, p = 0.622, n = 5F/5M; NGB-2904: t_(8)_ = 0.74, p = 0.475, n = 5F/5M; NOL long: vehicle t_(6)_ = 1.16, p = 0.289, n = 4F/4M; NGB-2904: t_(4)_ = 0.35, p = 0.743, n = 3F/3M; data analyses from Figure 3a). **(b)** No sex differences were detected in discrimination index evaluated by NOR or NOL in WT and D3-KO young mice that underwent a short or a long T1 (NOR short: WT t_(8)_ = 0.64, p = 0.536, n = 5F/5M; D3-KO: t_(8)_ = 0.58, p = 0.573, n = 5F/5M; NOR long: WT t_(6)_ = 1.61, p = 0.159, n = 4F/4M; D3-KO: t_(6)_ = 0.35, p = 0.736, n = 4F/4M; NOL short: WT t_(6)_ = 0.89, p = 0.404, n = 4F/4M; D3-KO: t_(6)_ = 0.48, p = 0.643, n = 4F/4M; NOL long: WT t_(7)_ = 0.1, p = 0.918, n = 4F/5M; D3-KO: t_(8)_ = 0.05, p = 0.961, n = 4F/6M; from Figure 3c). **(c)** No sex differences were detected in total exploration time evaluated during NOR or NOL in vehicle- and NGB-2904-treated young mice that underwent a short T1 (NOR: vehicle t_(6)_ = 0.36, p = 0.725; NGB-2904: t_(7)_ = 0.01, p = 0.99; NOL: vehicle t_(8)_ = 0.55, p = 0.594; NGB-2904: t_(8)_ = 0.31, p = 0.760; from Figure S4a) or a long T1 (NOR: vehicle t_(9)_ = 0.51, p = 0.619; NGB-2904: t_(10)_ = 1.42, p = 0.185; NOL: vehicle t_(6)_ = 0.23, p = 0.823; NGB-2904: t_(4)_ = 0.02, p = 0.981; from Figure S4c). **(d)** No sex differences were detected in total exploration time evaluated during NOR or NOL in WT and D3-KO young mice that underwent a short T1 (NOR: WT t_(8)_ = 1.46, p = 0.183; D3-KO: t_(8)_ = 2.01, p = 0.080; NOL: WT t_(6)_ = 1.03, p = 0.341; D3-KO: t_(6)_ = 0.608, p = 0.566; from Figure S4d) or a long T1 (NOR: WT t_(6)_ = 0.77, p = 0.467; D3-KO: t_(6)_ = 0.15, p = 0.880; NOL: WT t_(7)_ = 1.42, p = 0.197; D3-KO: t_(8)_ = 1.21, p = 0.259; from Figure S4e). **(e)** No sex differences were detected in the OF results testing the time spent in center of the arena (WT, vehicle: t_(7)_ = 1.38, p = 0.210, n = 4F/5M; WT, NGB-2904: t_(7)_ = 1.25, p = 0.250, n = 5F/4M; D3-KO: t_(7)_ = 0.26, p = 0.8, n = 5F/4M; from Figure S4f) and **(f)** the number of entries into the center (WT, vehicle: t_(7)_ = 1.93, p = 0.09; WT, NGB-2904: t_(7)_ = 0.13, p = 0.894; D3-KO: t_(7)_ = 0.64, p = 0.540; from Figure S4g). Data expressed as mean ± SEM. Veh = Vehicle; NGB = NGB-2904.


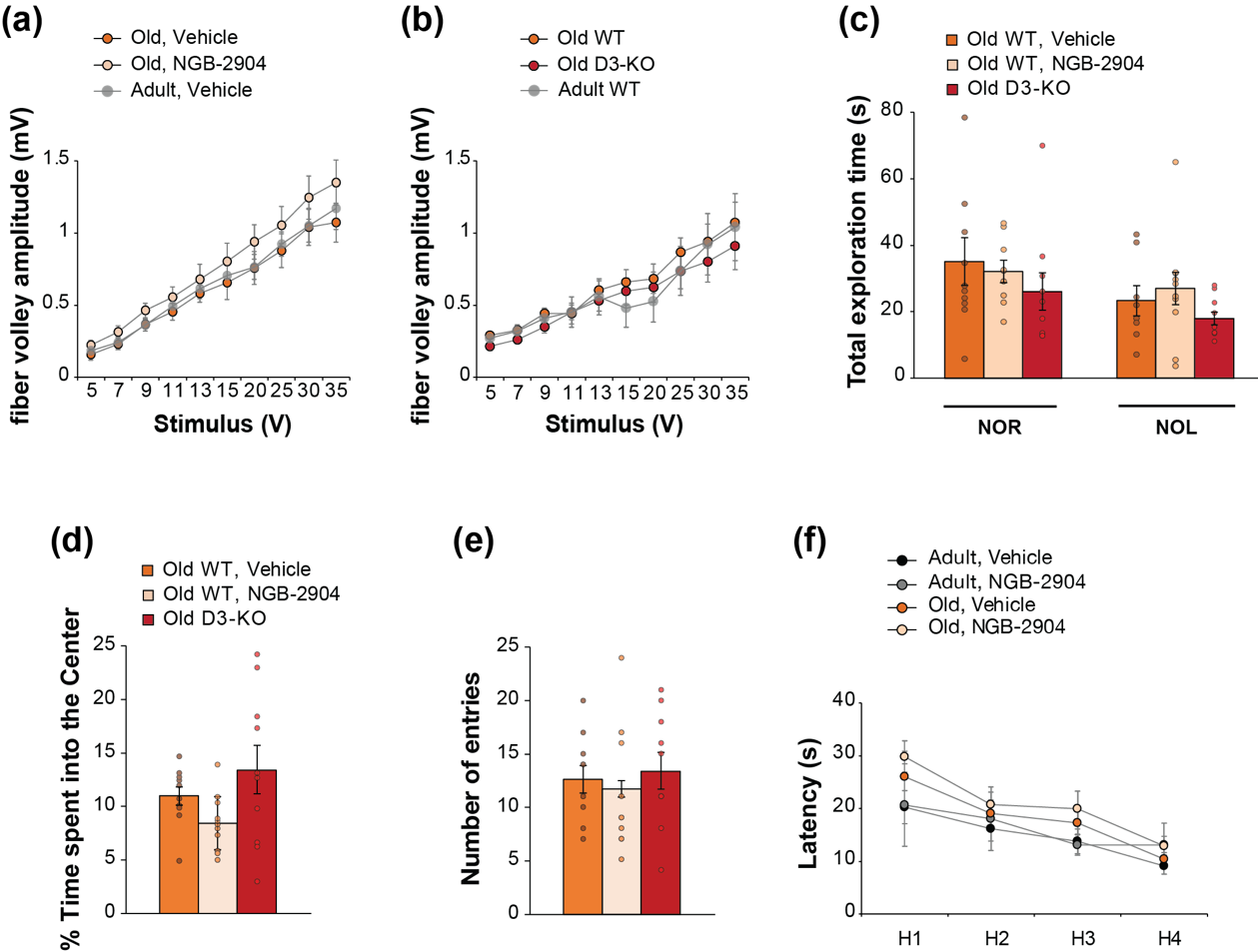


**FIGURE S6 – D3R blockade or genetic deletion did not modify presynaptic transmission, exploratory behavior and mobility in aged mice. (a)** No differences were found in the afferent volley amplitude assessed during basal synaptic transmission recordings between adult and old mice treated with NGB-2904 or vehicle (F_(2,30)_ = 0.944, p = 0.4). **(b)** Afferent volley was unchanged among adult WT, aged WT or aged D3-KO mice (F_(2,28)_ = 0.217, p = 0.806). **(c)** Total exploration time was not different among adult WT, aged WT or aged D3-KO mice in NOR (F_(2,26)_ = 0.57, p = 0.572) and NOL (F_(2,25)_ = 1.177, p = 0.325). **(d)** Open field test showed no differences in the three groups of mice (n = 10/11/10) in the time spent in the center of the arena (F_(2,28)_ = 3.069, p = 0.062) and **(e)** in the number of entries into the center (F_(2,28)_ = 0.274, p = 0.762). **(f)** No differences were found between adult and old mice treated with NGB-2904 or vehicle in the latency to reach the platform tested at the visible platform trial (F_(3,33)_ = 1.856, p = 0.156). Data expressed as mean ± SEM.

**
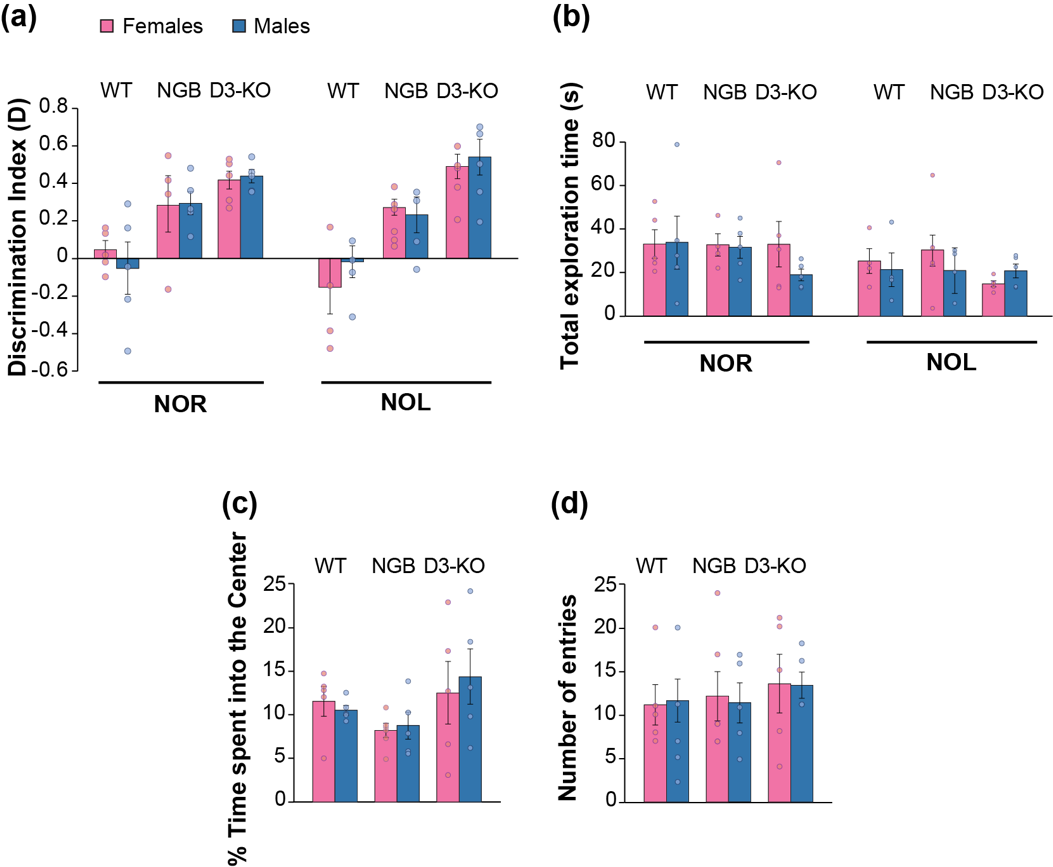
**

**FIGURE S7 – Sex did not influence the effects of D3R blockade in behavioral studies in old mice. (a)** No sex differences were detected in the discrimination index evaluated by NOR or NOL tests in vehicle- and NGB-2904-treated WT mice, as well as in D3-KO animals (NOR: WT t_(8)_ = 0.67, p = 0.519, n = 5F/5M; NGB-2904: t_(7)_ = 0.07, p = 0.944, n = 4F/5M; D3-KO: t_(8)_ = 0.33, p = 0.751, n = 5F/5M; NOL: WT t_(6)_ = 0.8, p = 0.452, n = 4F/4M; NGB-2904: t_(9)_ = 0.45, p = 0.662, n = 7F/4M; D3-KO: t_(8)_ = 0.43, p = 0.678, n = 5F/5M; data analyses from Figure 5e). **(b)** No sex differences were detected in total exploration time evaluated during NOR or NOL tests in vehicle- and NGB-2904-treated WT mice, as well as in D3-KO animals (NOR: WT t_(8)_ = 0.02, p = 0.984; NGB-2904: t_(7)_ = 0.15, p = 0.879; D3-KO: t_(8)_ = 1.31, p = 0.226; NOL: WT t_(6)_ = 0.4, p = 0.699; NGB-2904: t_(9)_ = 0.92, p = 0.378; D3-KO: t_(8)_ = 1.72, p = 0.124; data analyses from Figure S6c). **(c)** No sex differences were detected in the OF results testing the time spent in center of the arena (WT, vehicle: t_(8)_ = 0.57, p = 0.579, n = 5F/5M; WT, NGB-2904: t_(9)_ = 0.33, p = 0.745, n = 6F/5M; D3-KO: t_(8)_ = 0.38, p = 0.710, n = 5F/5M; from Figure S6d) and **(d)** the number of entries into the center (WT, vehicle: t_(8)_ = 1.19, p = 0.266; WT, NGB-2904: t_(9)_ = 0.2, p = 0.842; D3-KO: t_(8)_ = 0.05, p = 0.958; from Figure S6e). Data expressed as mean ± SEM.

| **Experimental Condition** | **WT+3T** | **WT+3T+NGB** | **D3-KO+3T** | **Statistical analyses** (P value) | | |
| --- | --- | --- | --- | --- | --- | --- |
| Number of asymmetric synapses (n) from 2 slices | 102 | 103 | 104 |  |  |  |
|  |  |  |  | **WT+3T**  ***vs***  **WT+3T+NGB** | **WT+3T**  ***vs***  **D3-KO+3T** | **WT+3T+NGB**  ***vs***  **D3-KO+3T** |
| Vesicle pool (n) | 57.45 ± 1.87 | 58.03 ± 2.01 | 61.87 ± 2.46 | P > 0.99 | P > 0.99 | P > 0.99 |
| Docked vesicles (n) | 2.01 ± 0.08 | 2.22 ± 0.08 | 2.27 ± 0.09 | P = 0.33 | P = 0.16 | P > 0.99 |
| Area of spines (µm^2^) | 0.14 ± 0.01 | 0.13 ± 0.008 | 0.13 ± 0.009 | P > 0.99 | P > 0.99 | P > 0.99 |
| **PSD length (µm)** | 0.191 ± 0.004 | 0.241 ± 0.005 | 0.217 ± 0.004 | **P < 0.0001** | **P = 0.0006** | **P = 0.0048** |
| Perforated PSD | 4.91 ± 2.14% | 7.76 ± 2.65% | 7.69 ± 2.62% | P > 0.99 | P > 0.99 | P > 0.99 |

**TABLE S1 –** Detailed results of electron microscopy performed on hippocampal slices recorded in the following experimental conditions: aged WT + 3 TBS (WT+3T), aged WT + 3 TBS in the presence of NGB-2904 (WT+3T+NGB), aged D3-KO + 3 TBS (D3-KO+3T). Statistical analyses performed by non-parametric Kruskal-Wallis test with Dunn’s test for multiple comparison. 3 TBS = strong tetanic stimulation. Number of microscopical fields: WT+3T (n = 99); WT+3T+NGB (n = 100); D3-KO+3T (n = 99). For all parameters values are mean ± SEM.
